# Supplementary material for: Vacancy-ordered perovskite superlattice in cerium titanate negative electrode for enhanced lithium-ion storage
Source: Nat Commun. 2025 Dec 12;16:11413. doi: 10.1038/s41467-025-66233-6 (PMC12738579; doi:10.1038/s41467-025-66233-6)
Supplement: Supplementary file 1 — Supplementary Information [file 41467_2025_66233_MOESM1_ESM.pdf]

# Supplementary Information

## Vacancy-ordered perovskite superlattice in cerium titanate negative electrode for enhanced lithium-ion storage

Xuhui Xiong[1], Zhengwang Liu[1], Ruixuan Zhang[2], Liting Yang[1], Guisheng Liang[1], Ke Pei[1], Renchao Che[1]*

[1]Laboratory of Advanced Materials, Shanghai Key Lab of Molecular Catalysis and Innovative Materials, State Key Laboratory of Coatings for Advanced Equipment, College of Smart Materials and Future Energy, Fudan University, Shanghai 200438, China

[2]Zhejiang Laboratory, Hangzhou 311100, China

*Corresponding author Email: rcche@fudan.edu.cn

# Table of Contents

**Table of Contents** ........................................................................................2

**Supplementary Notes**.....................................................................................3

**Supplementary Note 1** Evaluation of pseudocapacitive behavior. ......................3

**Supplementary Note 2** Quantification of pseudocapacitive contribution ratios. .4

**Supplementary Note 3** Calculation of $Li^+$ diffusion coefficients. .......................5

**Supplementary Figures** .....................................................................................6

**Supplementary Tables** ......................................................................................43

**Supplementary Table 1** XRD Rietveld refinement results of CTO. .................43

**Supplementary Table 2** Detailed comparison of rate performance among representative negative electrodes. ....................................................................44

**Supplementary Table 3** Detailed comparison of cycling performance among representative negative electrodes. ....................................................................45

**Supplementary Table 4** Detailed comparison of $Li^+$ diffusion coefficients among representative negative electrodes. ........................................................46

**Supplementary Table 5** Formation energies of different $Li^+$ storage sites in the tetragonal phase. ....................................................................................47

**Supplementary Table 6** Formation energies of different $Li^+$ storage sites in the pseudo-cubic phase. ...............................................................................48

**Supplementary References**..............................................................................49

## Supplementary Notes

**Supplementary Note 1** Evaluation of pseudocapacitive behavior.

The pseudocapacitive behavior of CTO was systematically evaluated based on the CV curves at varying scan rates. The relationship between the peak current ($i$) and the scan rate ($v$) can be described by the empirical power-law expression[1]:

$$i = a \cdot v^b \quad (1)$$

where $a$ and $b$ are fitting parameters, and the $b$-value is indicative of the underlying electrochemical kinetics. By taking the logarithm of both sides of equation (1), the relationship is linearized as follows[2]:

$$\log(i) = \log(a) + b \cdot \log(v) \quad (2)$$

By plotting $\log(i)$ versus $\log(v)$, the $b$-value can be determined from the slope of the fitting line. The $b$-value serves as a critical indicator of the dominant charge-storage mechanism[3]: a $b$-value of 0.5 suggests a diffusion-controlled process, a $b$-value of 1.0 indicates the surface-controlled process, and intermediate values of $0.5 < b < 1.0$ implies a mixed behavior involving both diffusion and pseudocapacitive contributions.

**Supplementary Note 2** Quantification of pseudocapacitive contribution ratios.

To quantify the relative contributions of pseudocapacitive and diffusion-controlled processes, the total current response in the CV curves can be separated into two components, as expressed in the following equation[4, 5]:

$$i(V) = k_1 \cdot v + k_2 \cdot v^{1/2} \tag{3}$$

In this formulation, $k_1 \cdot v$ represents the pseudocapacitive contribution, while $k_2 \cdot v^{1/2}$ accounts for the diffusion-controlled component. By further manipulating equation (3), the linearized form can be obtained as follows:

$$i(V)/v^{1/2} = k_1 \cdot v^{1/2} + k_2 \tag{4}$$

In this expression, $i(V)/v^{1/2}$ is plotted on the vertical axis, $v^{1/2}$ is plotted on the horizontal axis. The slope of the fitted line is the value of $k_1$, and the intercept corresponds to $k_2$. The pseudocapacitive contribution ratio can then be determined by calculating the relative magnitude of the pseudocapacitive term $k_1 \cdot v$ with respect to the total current.

**Supplementary Note 3** Calculation of $Li^+$ diffusion coefficients.

The $Li^+$ diffusion coefficients were determined using GITT, which is a widely adopted method for investigating ionic diffusion kinetics in electrodes. The calculation of $Li^+$ diffusion coefficients follows Fick's second law, expressed as follows[6, 7]:

$$D = \frac{4}{\pi\tau}(\frac{m_B V_m}{M_B S})^2(\frac{\Delta E_s}{\Delta E_\tau})^2 \tag{5}$$

In this equation, $\tau$ denotes the duration of a single current pulse, $m_B$ represents the active mass of the electrode, $M_B$ is the molar mass of the active material, $V_m$ is the molar volume of the active material, and $S$ is the surface area of the electrode. The terms $\Delta E_\tau$ and $\Delta E_s$ correspond to the potential changes during a single current pulse and the steady-state potential difference between two adjacent equilibrium states, respectively.

## Supplementary Figures

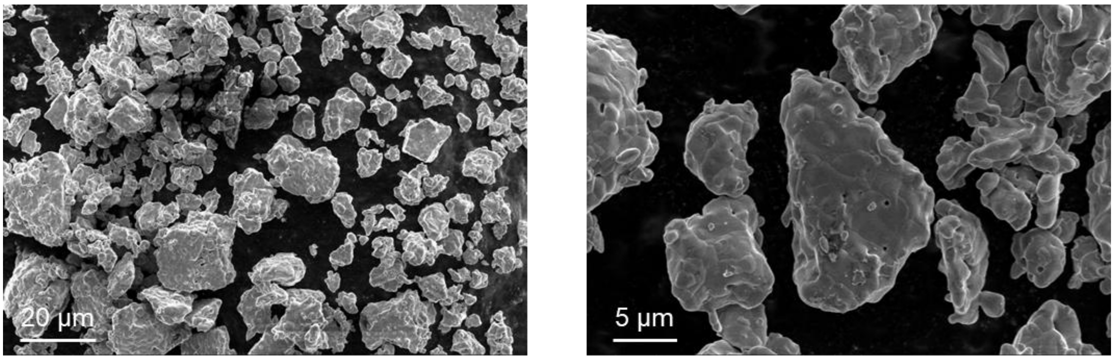


**Supplementary Fig. 1** SEM images of the synthesized CTO particles, showing their morphological and size characteristics.

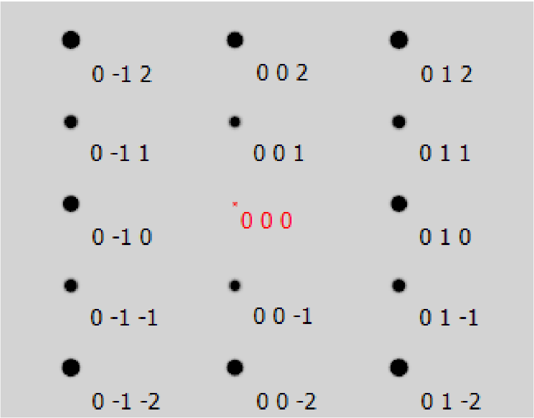


**Supplementary Fig. 2** Simulated electron diffraction patterns of CTO along the [100] zone axis.

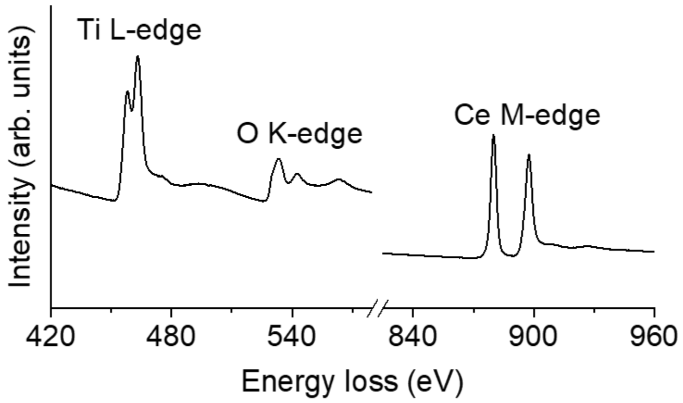


**Supplementary Fig. 3** EELS spectrum of CTO, showing characteristic peaks corresponding to the Ti L-edge, O K-edge, and Ce M-edge signals.

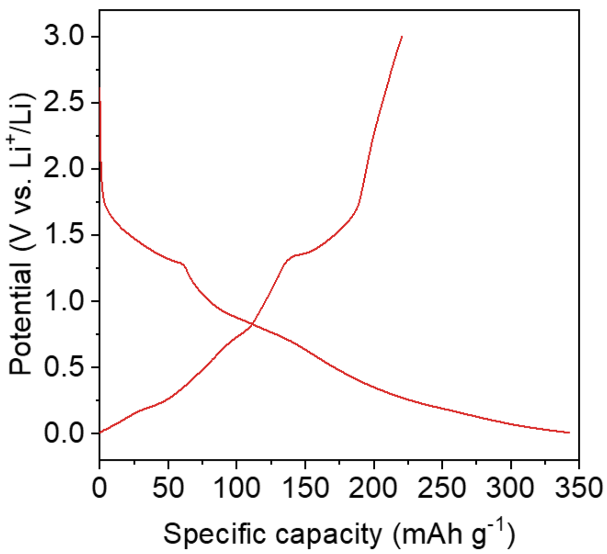


**Supplementary Fig. 4** Initial discharge/charge curves at a rate of 0.1 C (25 mA $g^{-1}$).

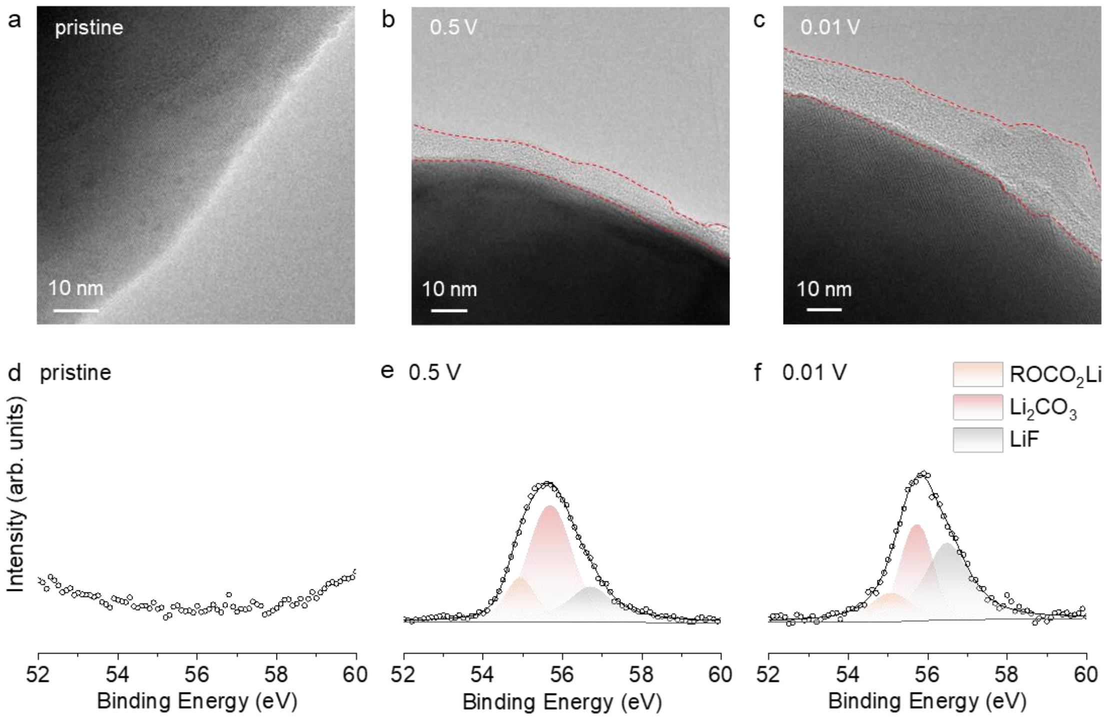


**Supplementary Fig. 5** Evolution of SEI layer on CTO during initial lithiation. **a-c** TEM images of CTO particle surfaces at different discharge states during the first cycle at 0.1 C (25 mA $g^{-1}$) and 25 °C: pristine state (**a**), 0.5 V (**b**), and 0.01 V (**c**), showing the formation and growth of the SEI layer. **d-f** Corresponding high-resolution Li 1*s* XPS spectra, revealing the chemical evolution of Li-containing species during lithiation.

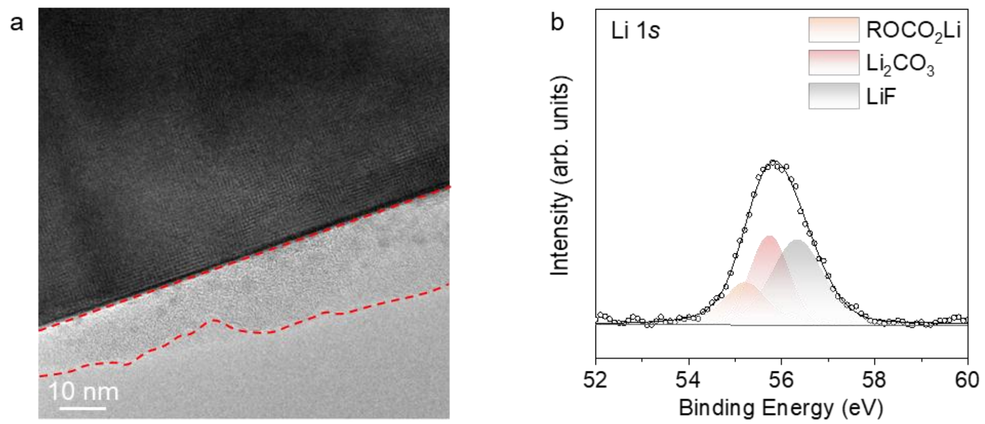


**Supplementary Fig. 6 a** TEM image of the CTO electrode after 2000 cycles at 10 C (2.5 A $g^{-1}$) and 25 °C, measured at ~100% state of discharge. **b** Corresponding high-resolution Li 1*s* XPS spectrum.

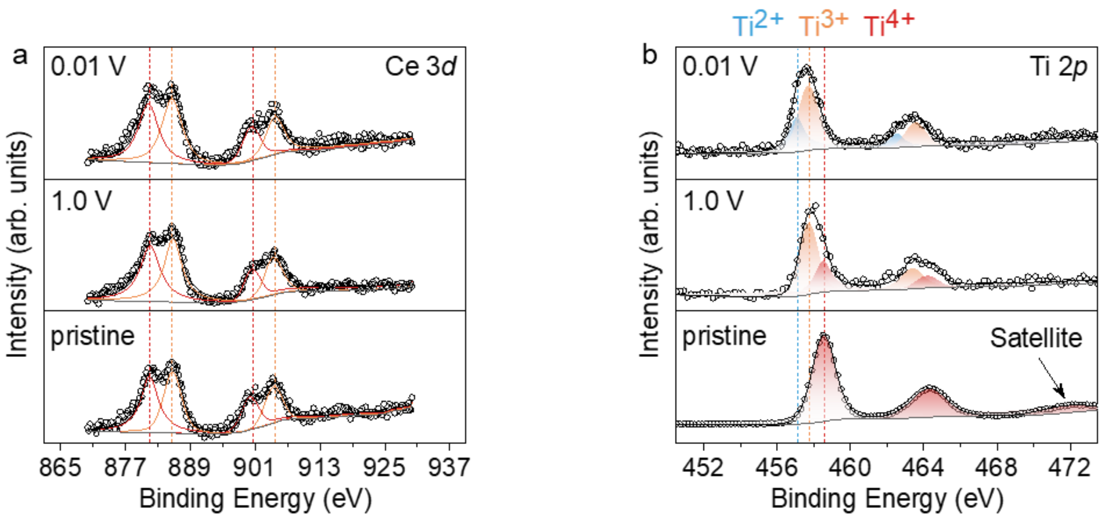


**Supplementary Fig. 7** XPS spectra of CTO at different discharge states during the first cycle at 0.1 C (25 mA $g^{-1}$) and 25 °C: pristine state, 1.0 V, and 0.01 V. **a** Evolution of Ce 3*d* high-resolution spectra. **b** Evolution of Ti 2*p* high-resolution spectra.

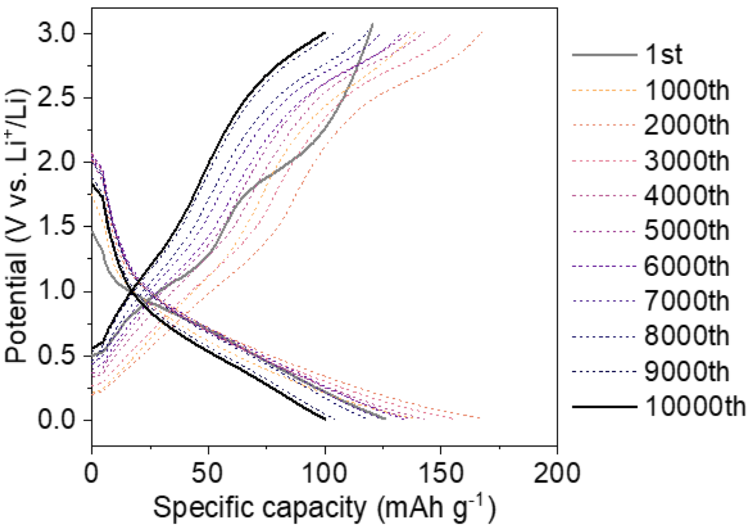


**Supplementary Fig. 8** Detailed discharge/charge curves of CTO during long-term cycling at 20 C (5.0 A $g^{-1}$).

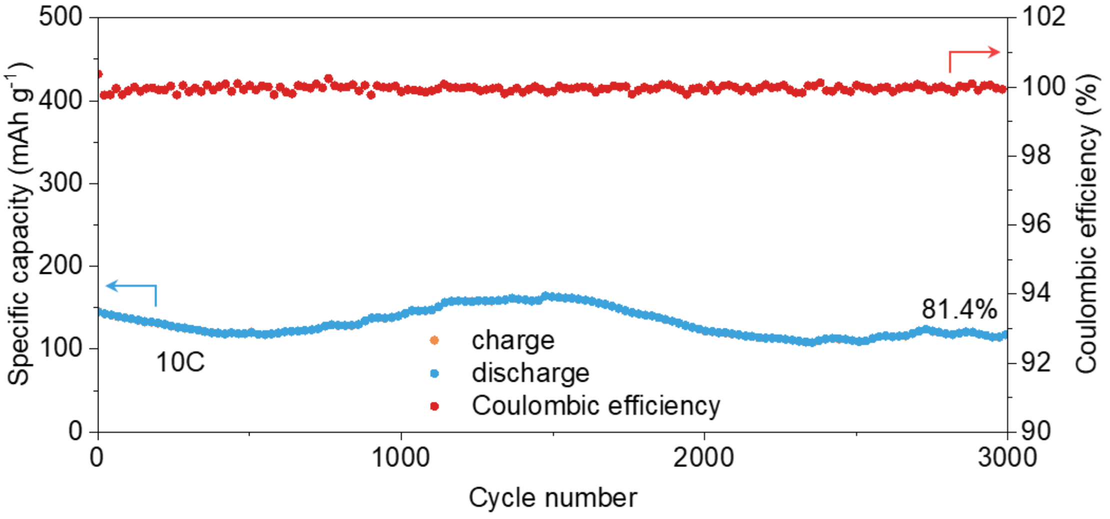


**Supplementary Fig. 9** Long-term cycling performance of the CTO electrode at a rate of 10 C (2.5 A $g^{-1}$).

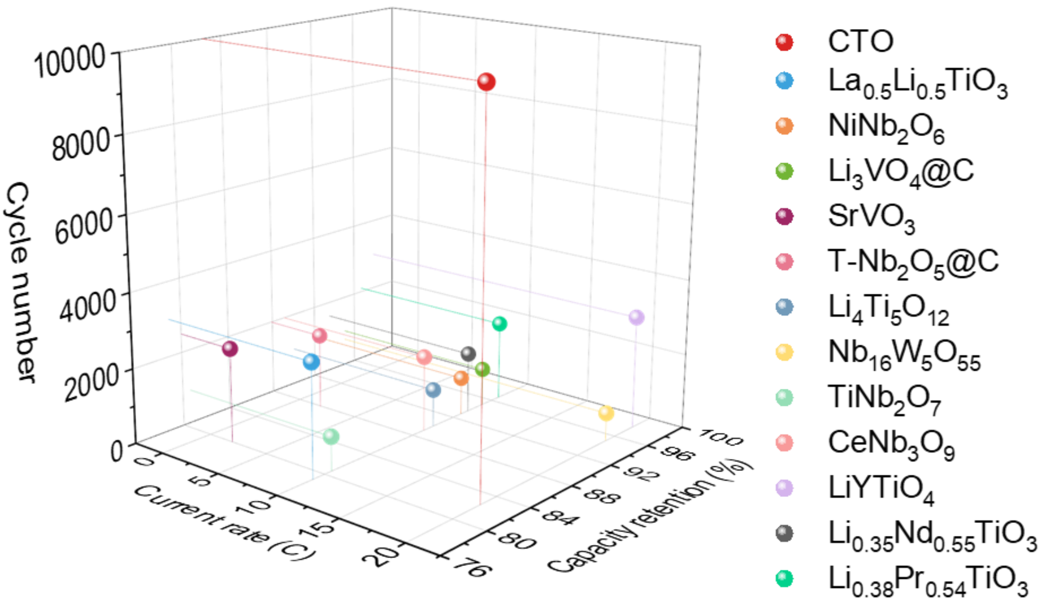


**Supplementary Fig. 10** Comparison of the cycling performance of CTO with representative intercalation-type negative electrodes, including $La_{0.5}Li_{0.5}TiO_3$ (1 C= 250 mA $g^{-1}$)[8], $NiNb_2O_6$ (1 C= 250 mA $g^{-1}$)[9], $Li_3VO_4$@C (1 C= 400 mA $g^{-1}$)[10], $SrVO_3$ (1 C= 1000 mA $g^{-1}$)[11], T-$Nb_2O_5$@C (1 C= 1000 mA $g^{-1}$)[12], $Li_4Ti_5O_{12}$ (1 C= 150 mA $g^{-1}$)[13], $Nb_{16}W_5O_{55}$ (1 C= 150 mA $g^{-1}$)[14], $TiNb_2O_7$ (1 C= 300 mA $g^{-1}$)[15], $CeNb_3O_9$ (1 C= 250 mA $g^{-1}$)[16], $LiYTiO_4$ (1 C= 200 mA $g^{-1}$)[17], $Li_{0.35}Nd_{0.55}TiO_3$ (1 C= 200 mA $g^{-1}$)[18], and $Li_{0.38}Pr_{0.54}TiO_3$ (1 C= 200 mA $g^{-1}$)[19]. A detailed summary is provided in Supplementary Table 3.

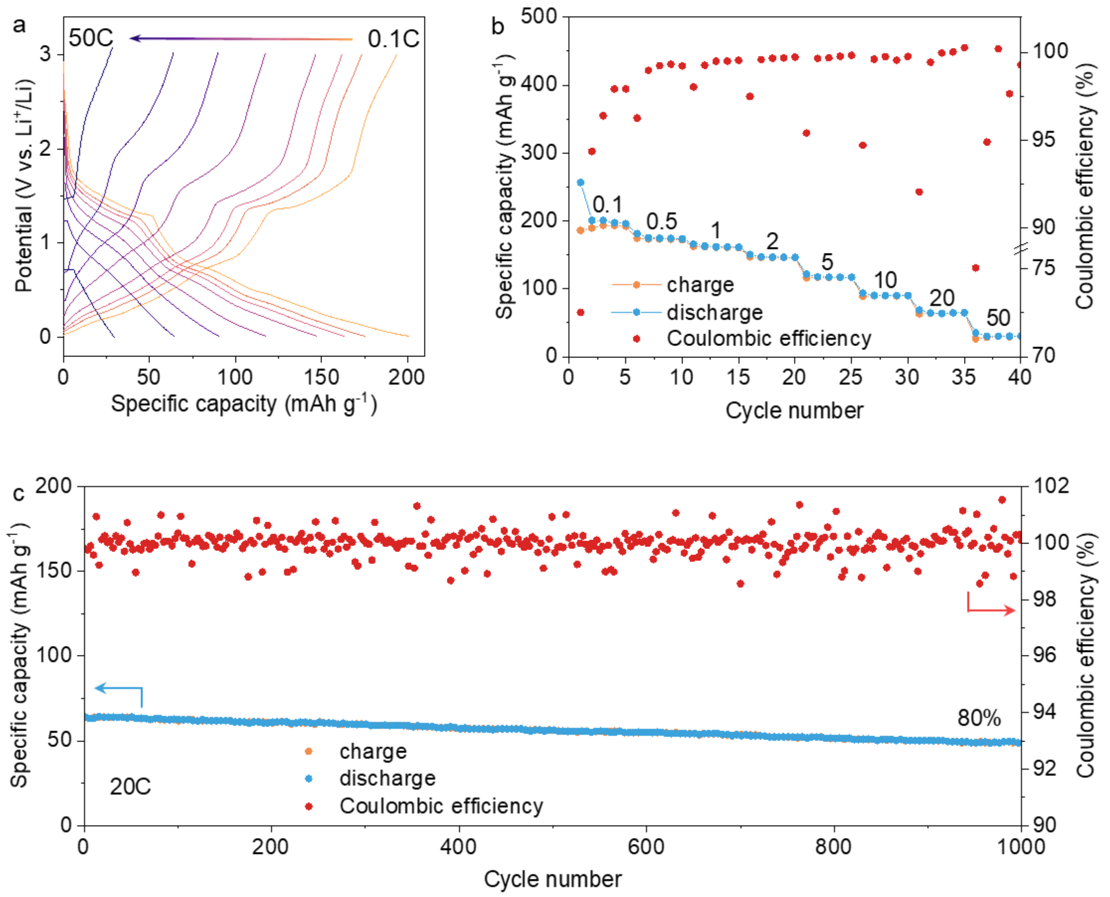


**Supplementary Fig. 11** Electrochemical performance of CTO electrode with a high mass loading of ~10.2 mg $cm^{-2}$. **a** Charge-discharge profiles at various current rates from 0.1 C to 50 C (1 C = 250 mA $g^{-1}$). **b** Rate capability and corresponding coulombic efficiencies at 0.1, 0.5, 1, 2, 5, 10, 20, and 50 C. **c** Cycling performance at a high rate of 20 C.

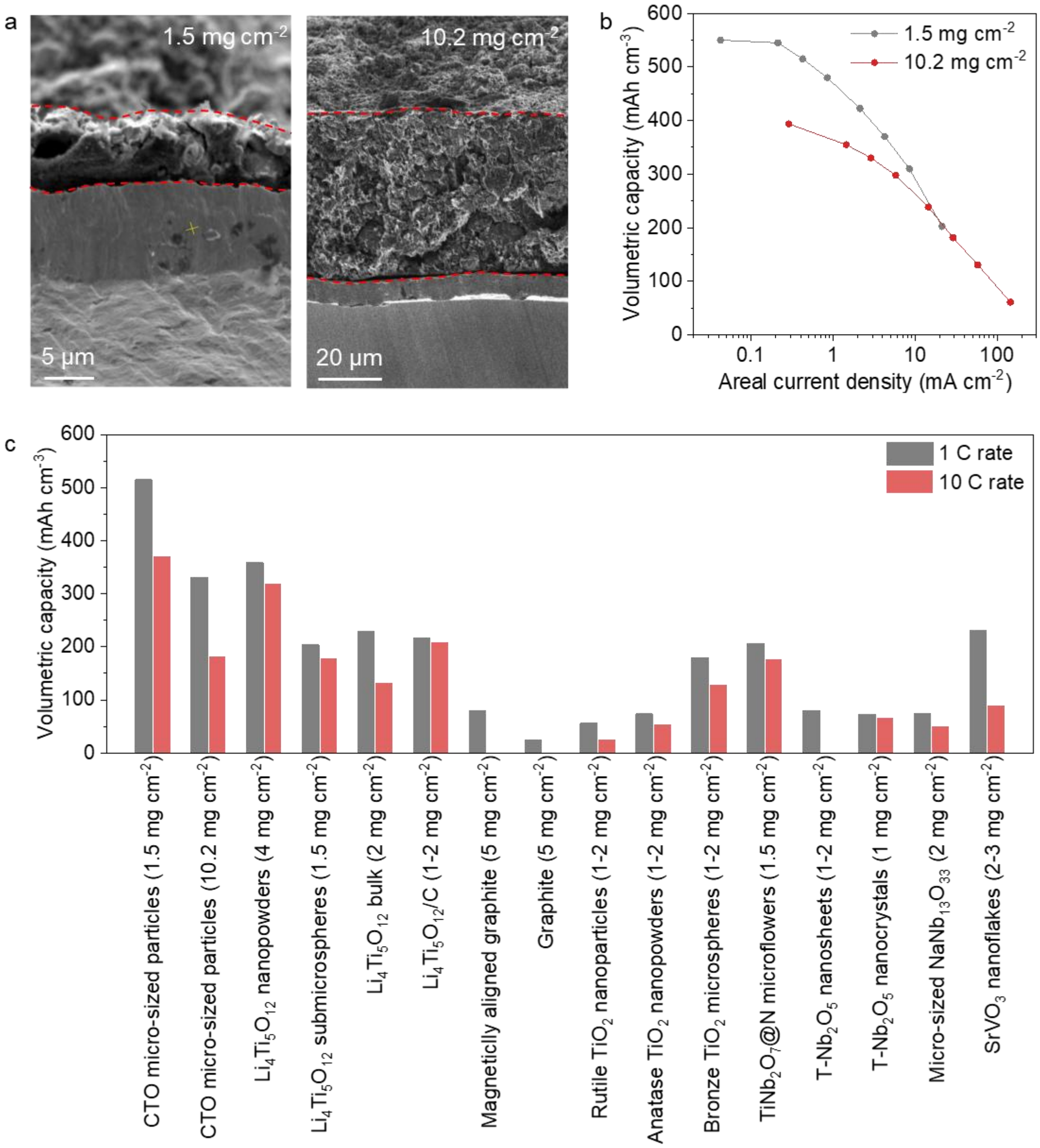


**Supplementary Fig. 12** Volumetric performance of CTO electrodes with low (~1.5 mg $cm^{-2}$) and high (~10.2 mg $cm^{-2}$) mass loadings. **a** Cross-sectional SEM images of CTO electrodes at the pristine state, prior to electrochemical cycling. **b** Areal current density versus volumetric capacity. **c** Comparison of volumetric capacities at 1 C (250 mA $g^{-1}$) and 10 C (2.5 A $g^{-1}$) with those of previously reported intercalation-type negative electrodes[11, 20, 21, 22, 23, 24, 25, 26, 27, 28, 29, 30, 31].

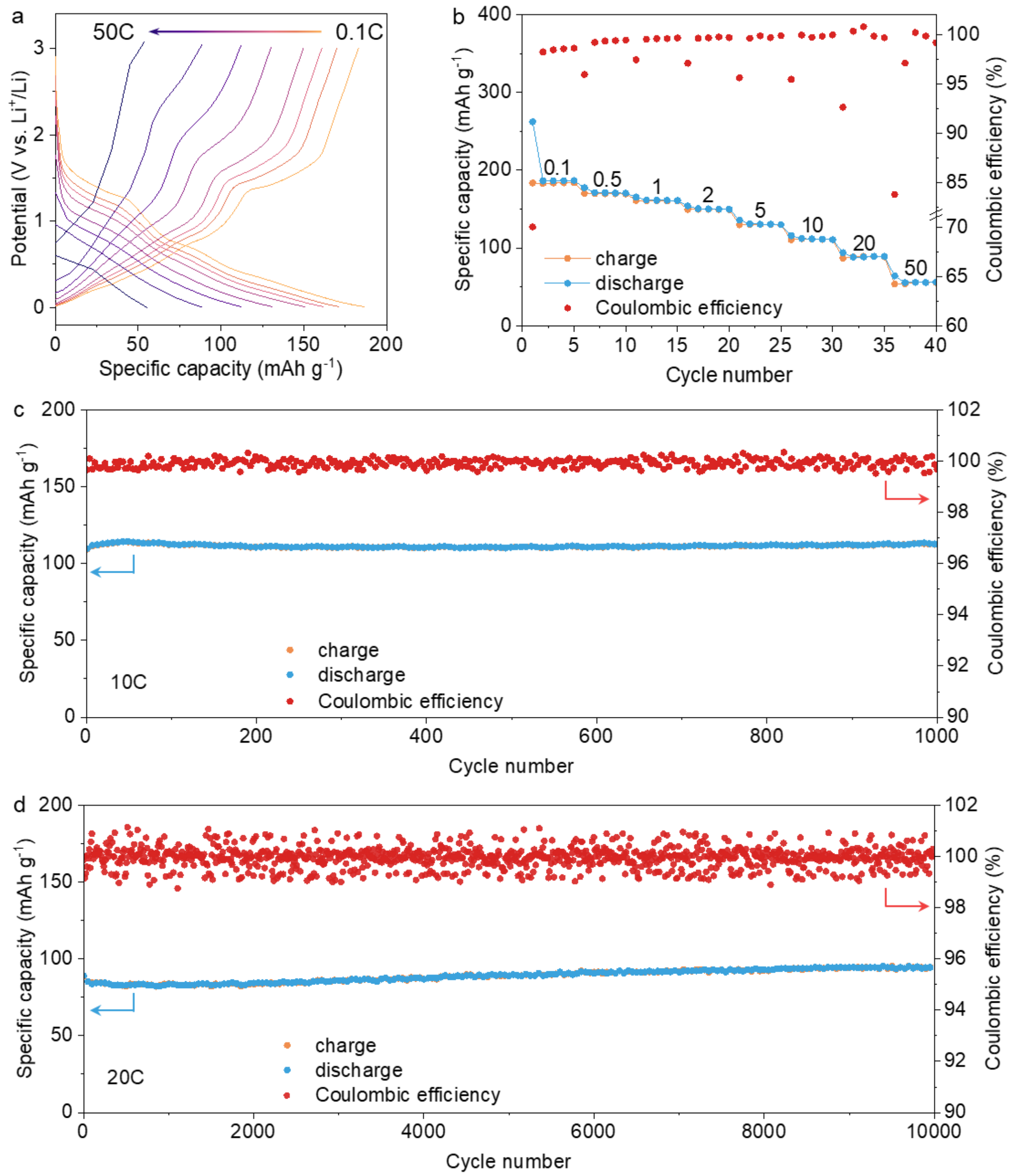


**Supplementary Fig. 13** Low-temperature electrochemical performance of the CTO electrode at 0 °C **a** Charge-discharge profiles at various current rates from 0.1 C to 50 C (1 C = 250 mA $g^{-1}$). **b** Rate capability and corresponding coulombic efficiencies at 0.1, 0.5, 1, 2, 5, 10, 20, and 50 C. **c** Cycling performance at 10 C. **d** Cycling performance at 20 C.

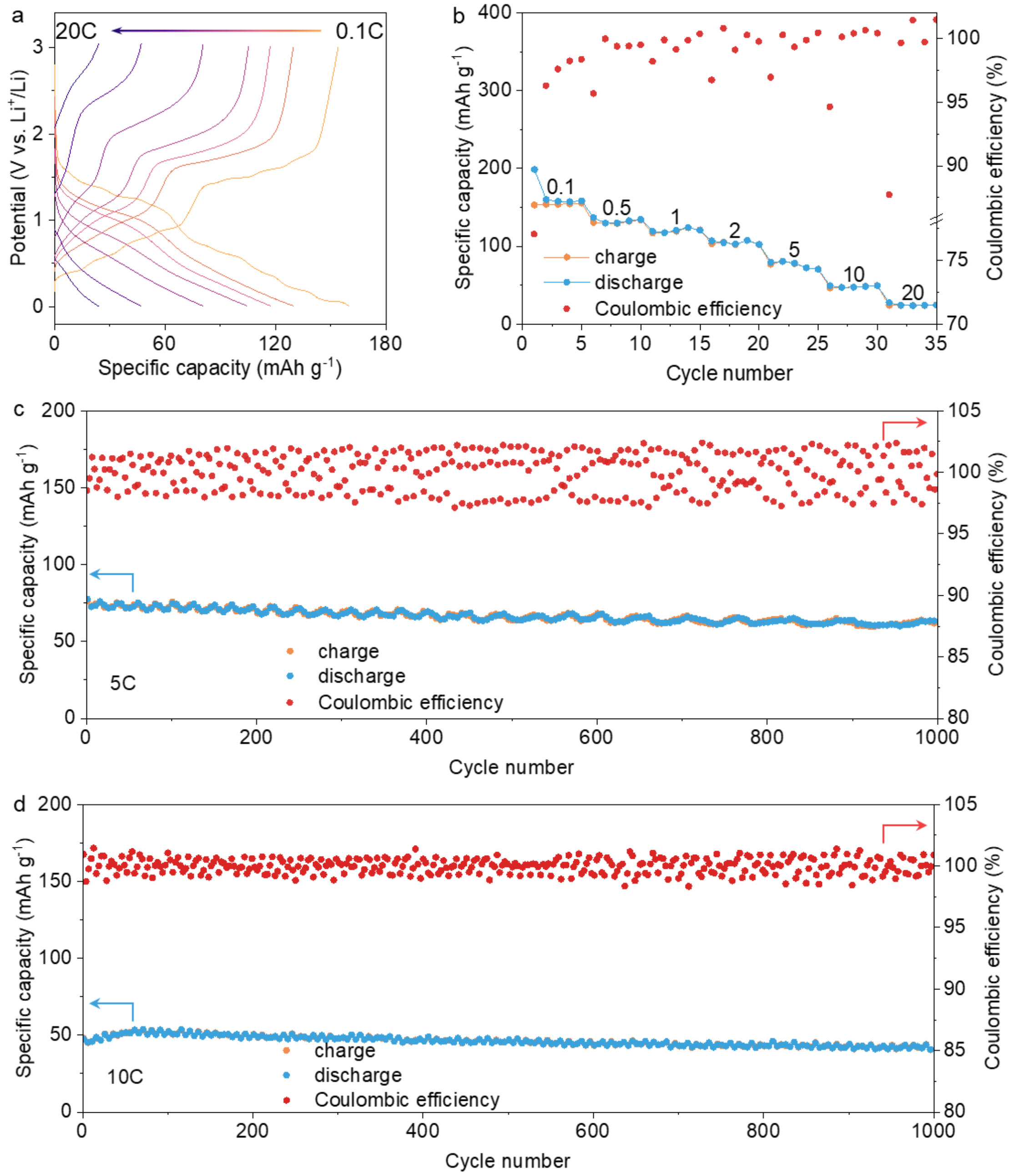


**Supplementary Fig. 14** Low-temperature electrochemical performance of the CTO electrode at -15 °C **a** Charge-discharge profiles at various current rates from 0.1 C to 20 C (1 C = 250 mA $g^{-1}$). **b** Rate capability and corresponding coulombic efficiencies at 0.1, 0.5, 1, 2, 5, 10, and 20 C. **c** Cycling performance at 5 C. **d** Cycling performance at 10 C

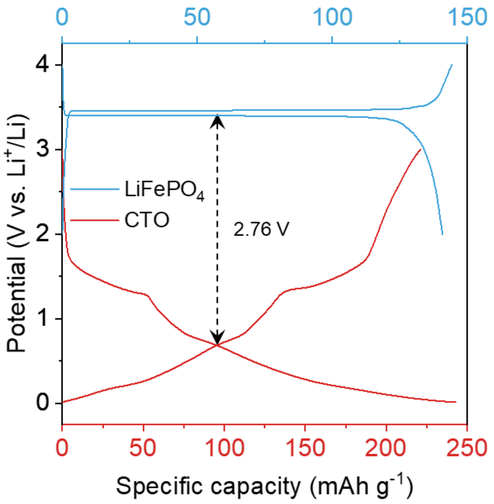


**Supplementary Fig. 15** Comparative charge/discharge profiles of $LiFePO_4$ and CTO electrodes.

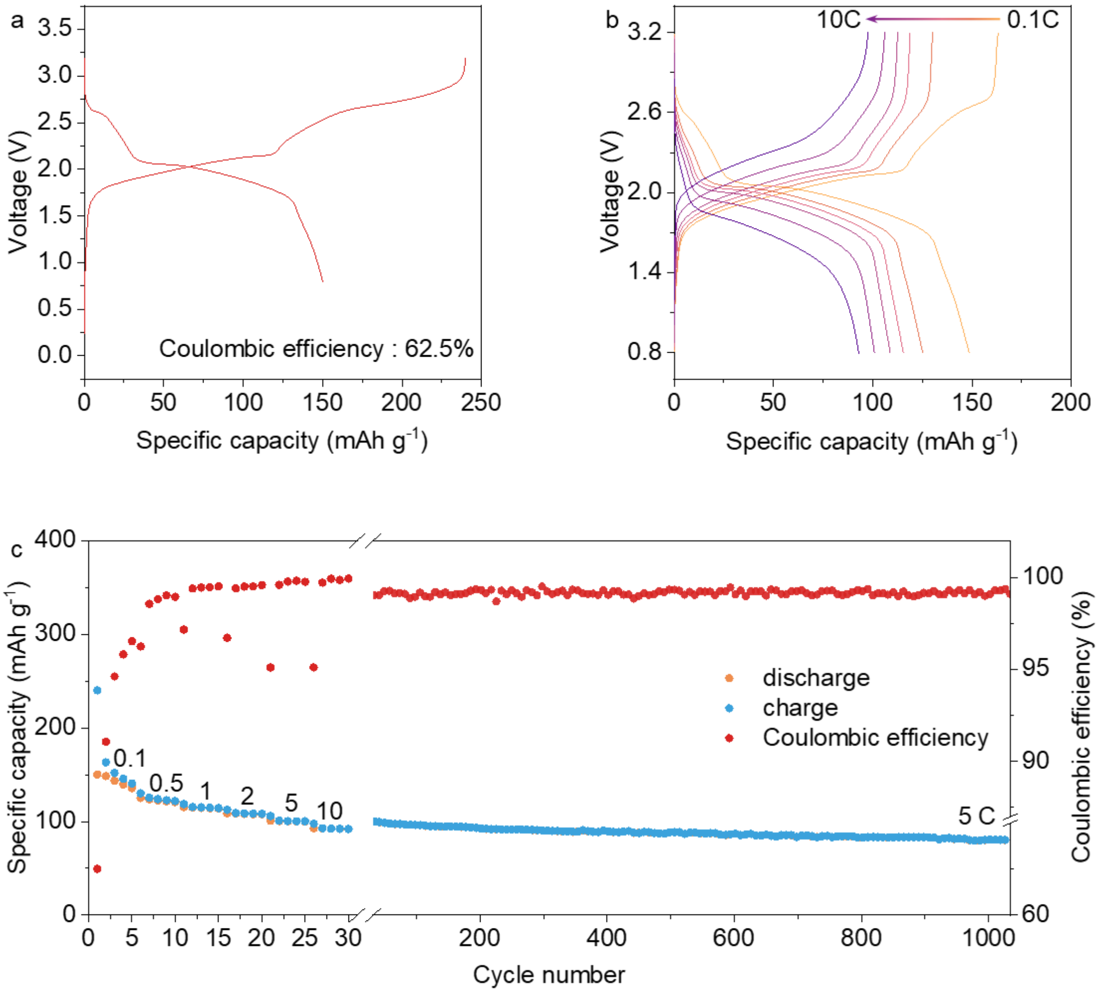


**Supplementary Fig. 16** Electrochemical performance of CTO||$LiFePO_4$ full cells. **a** Initial charge/discharge curves showing a first-cycle coulombic efficiency of 62.5%. **b** Charge/discharge profiles at different current rates from 0.1 C to 10 C (1 C = 250 mA $g^{-1}$). **c** Rate capability and long-term cycling performance at 5 C.

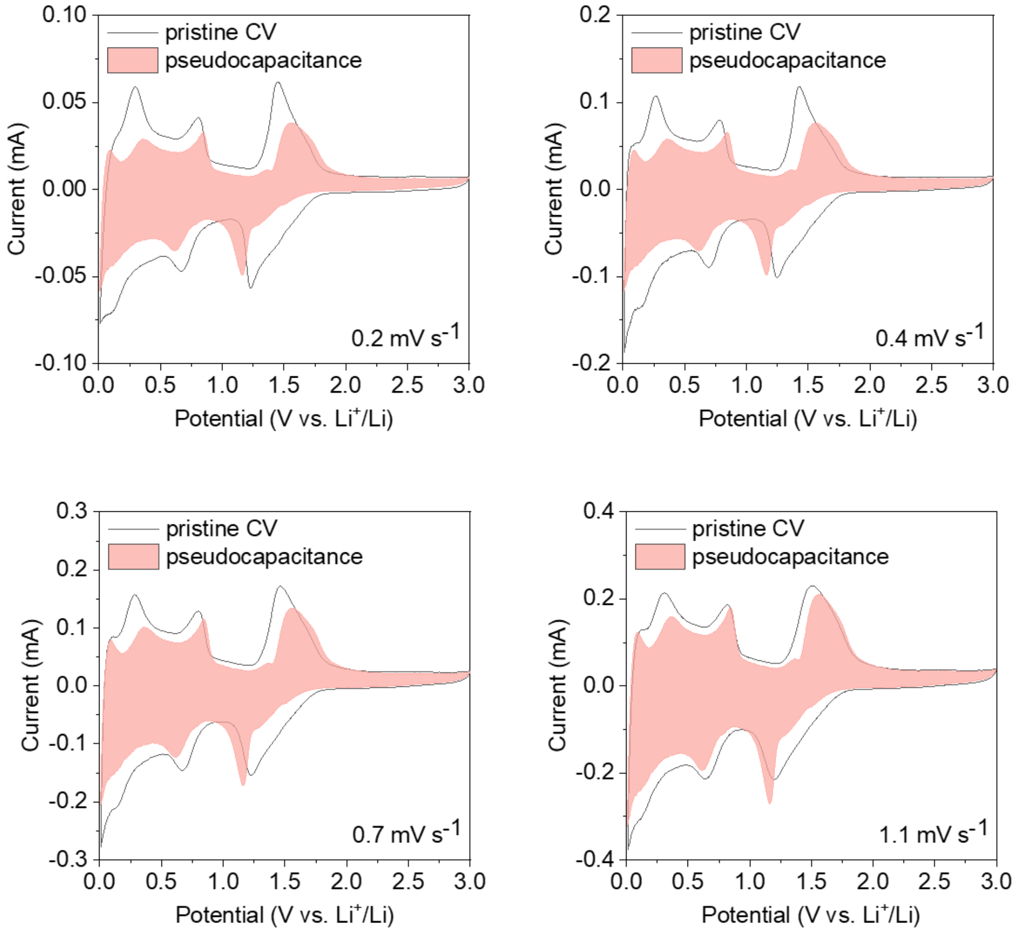


**Supplementary Fig. 17** Deconvolution of pseudocapacitive contributions in the CV curves at scan rates of 0.2, 0.4, 0.7, and 1.1 mV $s^{-1}$.

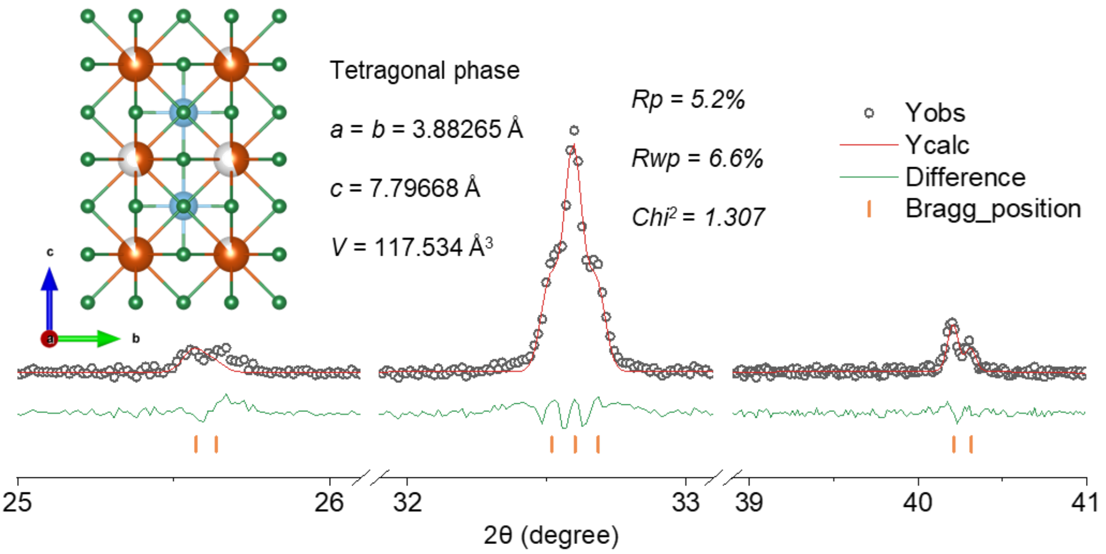


**Supplementary Fig. 18** Rietveld refinement of the XRD pattern for the pristine CTO electrode, prior to any electrochemical measurements. Orange, blue, and green spheres denote Ce, Ti, and O atoms, respectively.

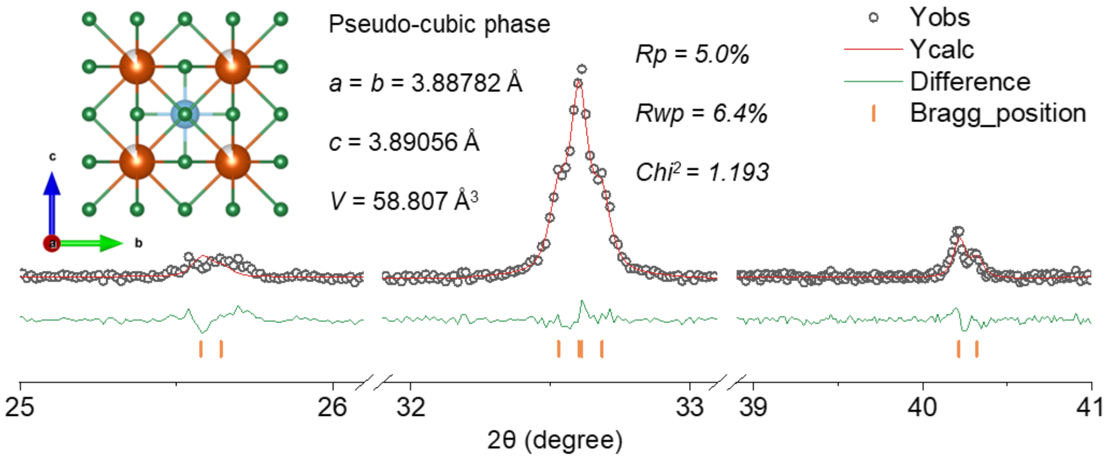


**Supplementary Fig. 19** Rietveld refinement of the XRD pattern for the CTO electrode after the first discharge to below 1.25 V at 0.5 C (125 mA $g^{-1}$) and 25 °C. Orange, blue, and green spheres denote Ce, Ti, and O atoms, respectively.

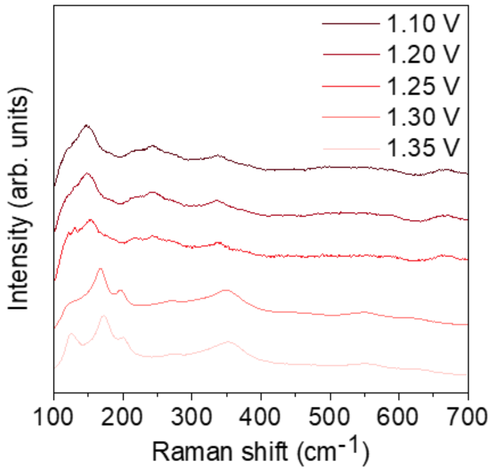


**Supplementary Fig. 20** Ex situ Raman spectra of CTO across the phase transition region. Raman spectra were collected after discharging to a series of potentials (1.35 V, 1.30 V, 1.25 V, 1.20 V, and 1.10 V) during the first cycle under 0.1 C (25 mA $g^{-1}$) and 25 °C to track structural evolution during lithiation. As the discharge proceeds, the main peaks at ~172 and ~354 $cm^{-1}$ gradually shift to lower wavenumbers, while shoulder peaks at ~124 and ~200 $cm^{-1}$ progressively weaken and merge. Meanwhile, the intensities of modes at ~242 and ~664 $cm^{-1}$ increase below 1.30 V. These spectral changes, particularly the progressive merging of low-frequency modes in the 100-200 $cm^{-1}$ region, indicate a gradual increase in structural symmetry, consistent with the transition from a tetragonal phase to a pseudo-cubic phase with higher symmetry.

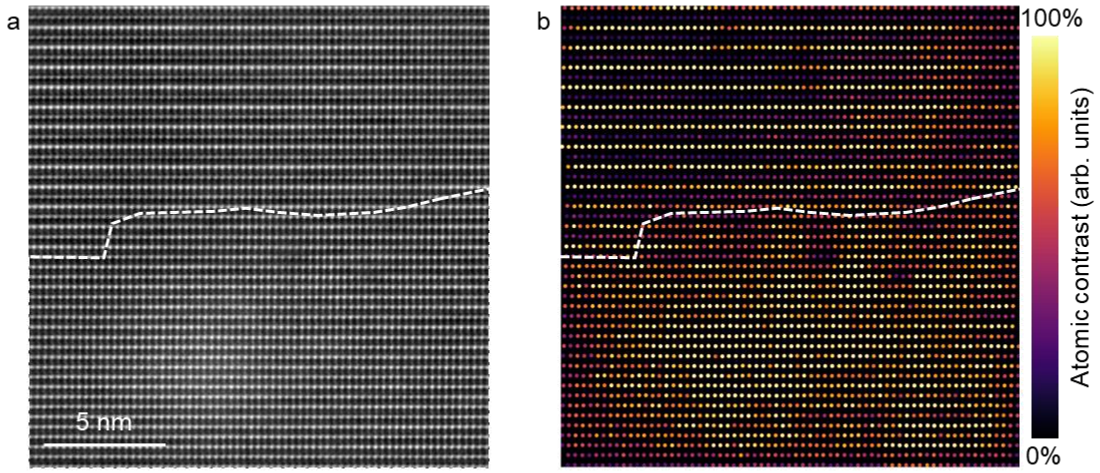


**Supplementary Fig. 21 a** HAADF-STEM image of CTO after discharging to 1.25 V during the first cycle under 0.1 C (25 mA $g^{-1}$) and 25 °C. **b** Reconstructed atomic contrast heatmap derived from the raw HAADF-STEM image. The phase boundary between the tetragonal and pseudo-cubic phases is marked by white dash lines.

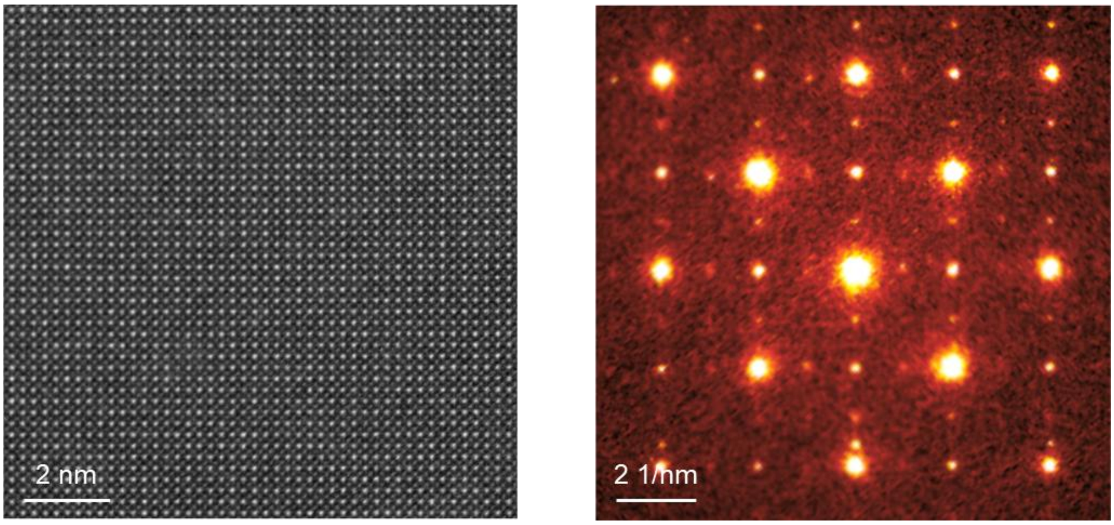


**Supplementary Fig. 22** HAADF-STEM and SAED images of CTO after topological phase transformation, viewed along the [100] zone axis. The electrode was harvested after discharging to 0.01 V during the first cycle under 0.1 C (25 mA $g^{-1}$) and 25 °C.

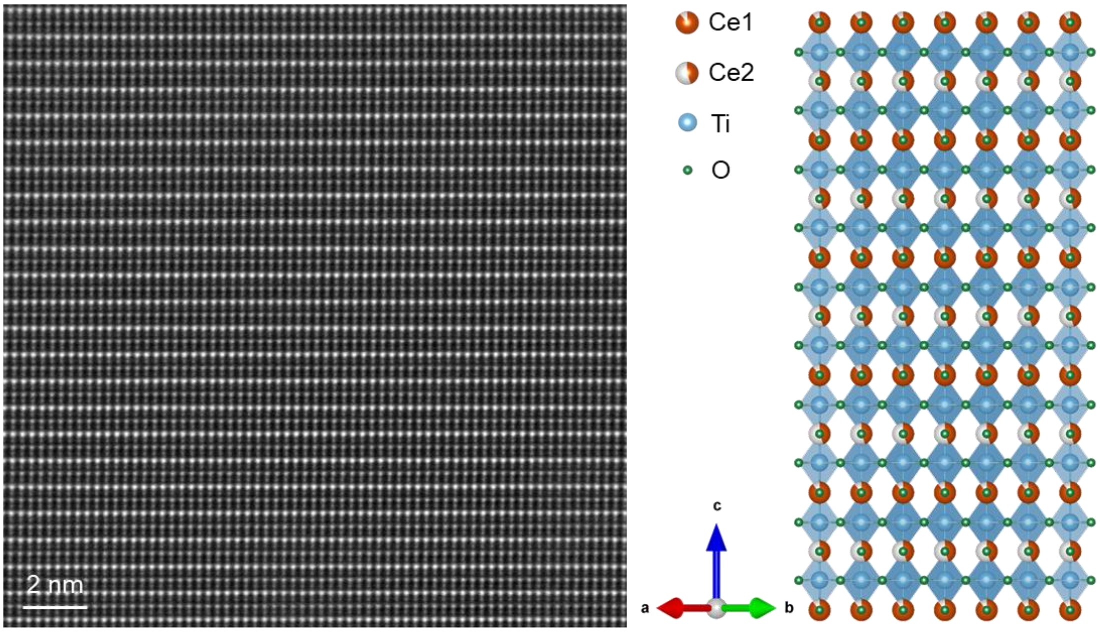


**Supplementary Fig. 23** HAADF-STEM image along the [110] zone axis of CTO after recharging to 3.0 V in the first cycle under 0.1 C (25 mA $g^{-1}$) and 25 °C, suggesting the recovery of vacancy-ordered superlattice structure upon delithiation.

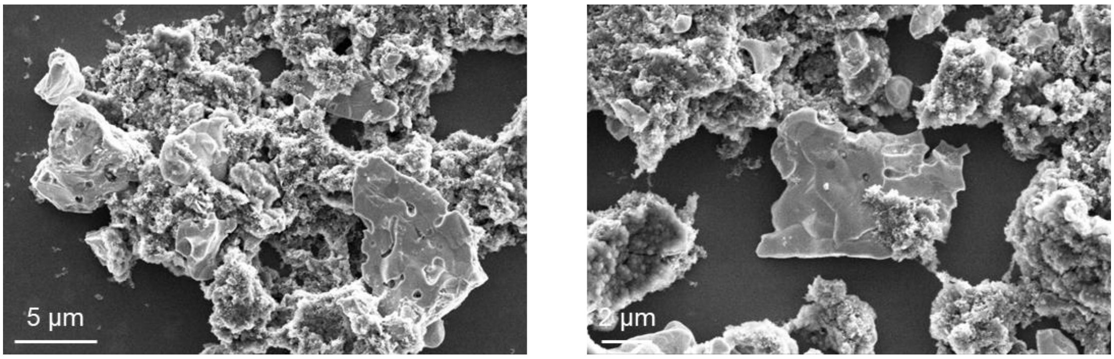


**Supplementary Fig. 24** SEM images of the CTO electrode after long-term cycling, highlighting the morphological preservation and particle integrity after prolonged cycling. The electrode was harvested after 10000 cycles under 20 C (5.0 A $g^{-1}$) and 25 ℃.

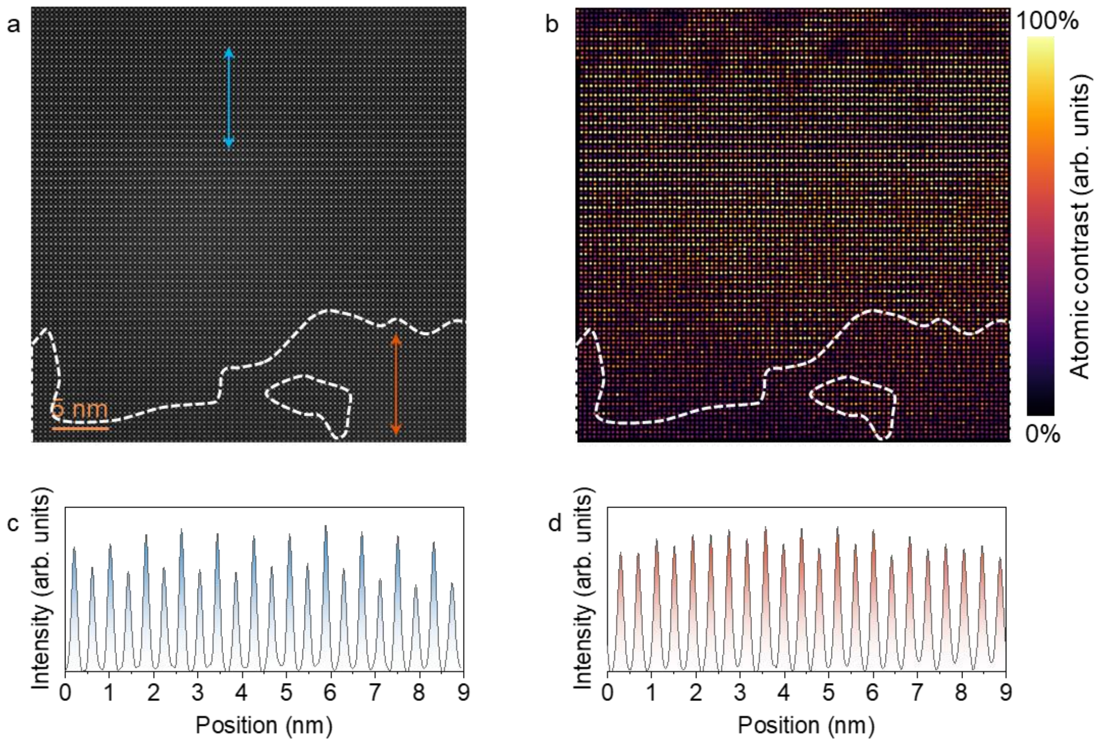


**Supplementary Fig. 25** **a** HAADF-STEM image along the [100] zone axis of CTO after long-term cycling. The electrode was harvested after 10000 cycles under 20 C (5.0 A $g^{-1}$) and 25 °C. The boundary between tetragonal and pseudo-cubic phases is indicated by white dash lines. **b** Reconstructed atomic contrast heatmap derived from the raw HAADF-STEM image, highlighting the coexistence of tetragonal and pseudo-cubic phases. **c** Line intensity profile along the blue line in the tetragonal region, showing pronounced periodic modulation of Ce atomic column intensities. **d** Line intensity profile along the orange line in the pseudo-cubic phase, where the contrast variation of Ce atomic columns is largely diminished.

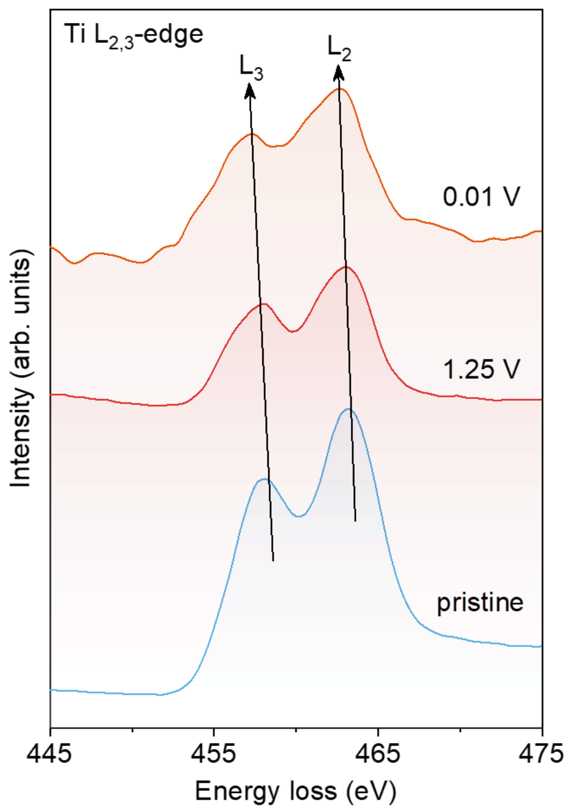


**Supplementary Fig. 26** EELS spectra of Ti $L_{2,3}$-edge signals at different lithiation states, illustrating the evolution of the Ti valence during lithiation. The electrodes were harvested at selected potentials (pristine, 1.25 V, and 0.01 V) during the first cycle under 0.1 C (25 mA $g^{-1}$) and 25 °C.

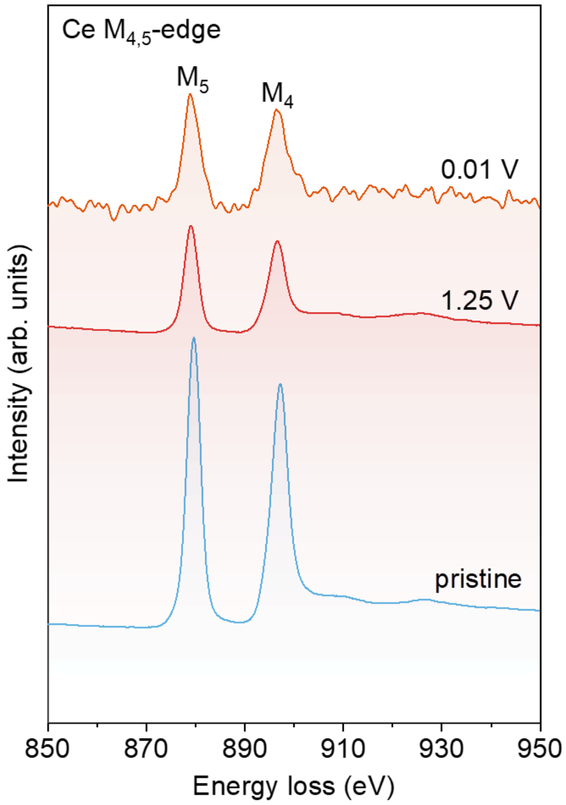


**Supplementary Fig. 27** EELS spectra of Ce $M_{4,5}$-edge signals at different lithiation states, where the peak positions remain unchanged, with only intensity variations observed during lithiation. The electrodes were harvested at selected potentials (pristine, 1.25 V, and 0.01 V) during the first cycle under 0.1 C (25 mA $g^{-1}$) and 25 °C.

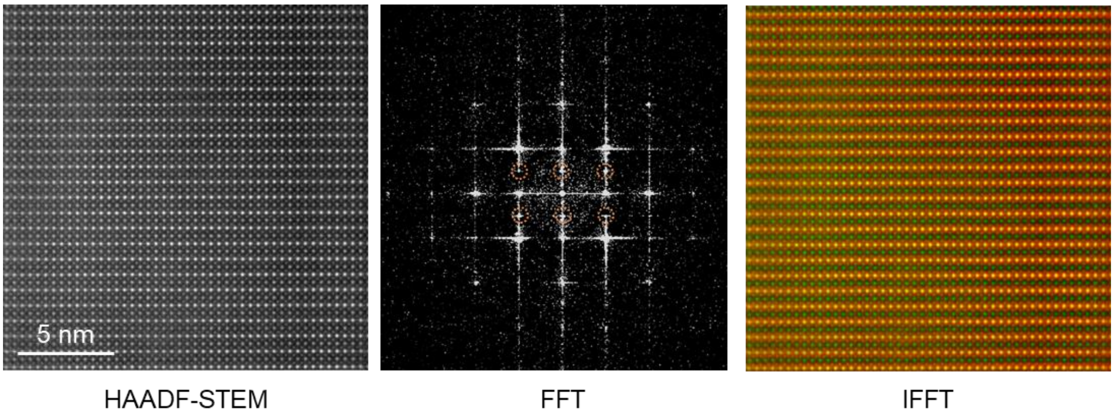


**Supplementary Fig. 28** Long-range ordered superlattice structure along the [100] zone axis of pristine CTO, which was collected prior to electrochemical measurements.

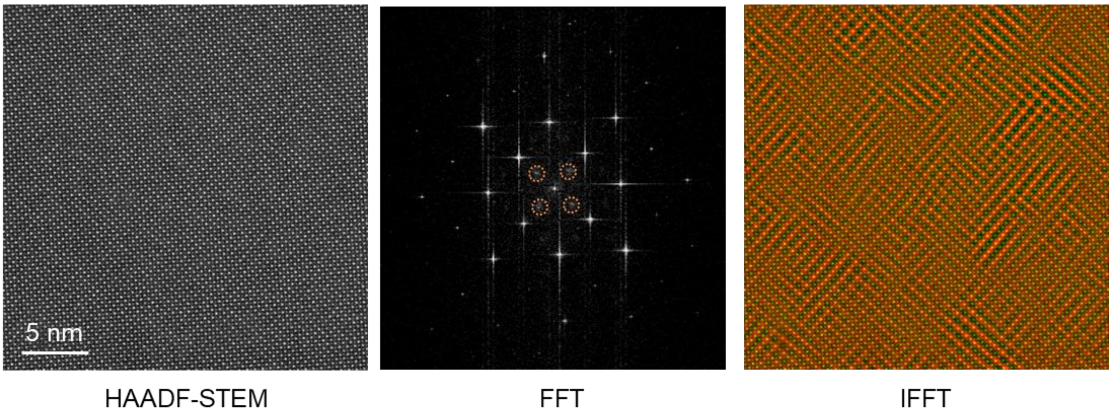


**Supplementary Fig. 29** Short-range ordered pseudo-cubic structure along the [100] zone axis of CTO after lithiation, which was collected after discharging to 0.01 V during the first cycle under 0.1 C (25 mA $g^{-1}$) and 25 °C, revealing the structural transformation induced by $Li^{+}$ insertion.

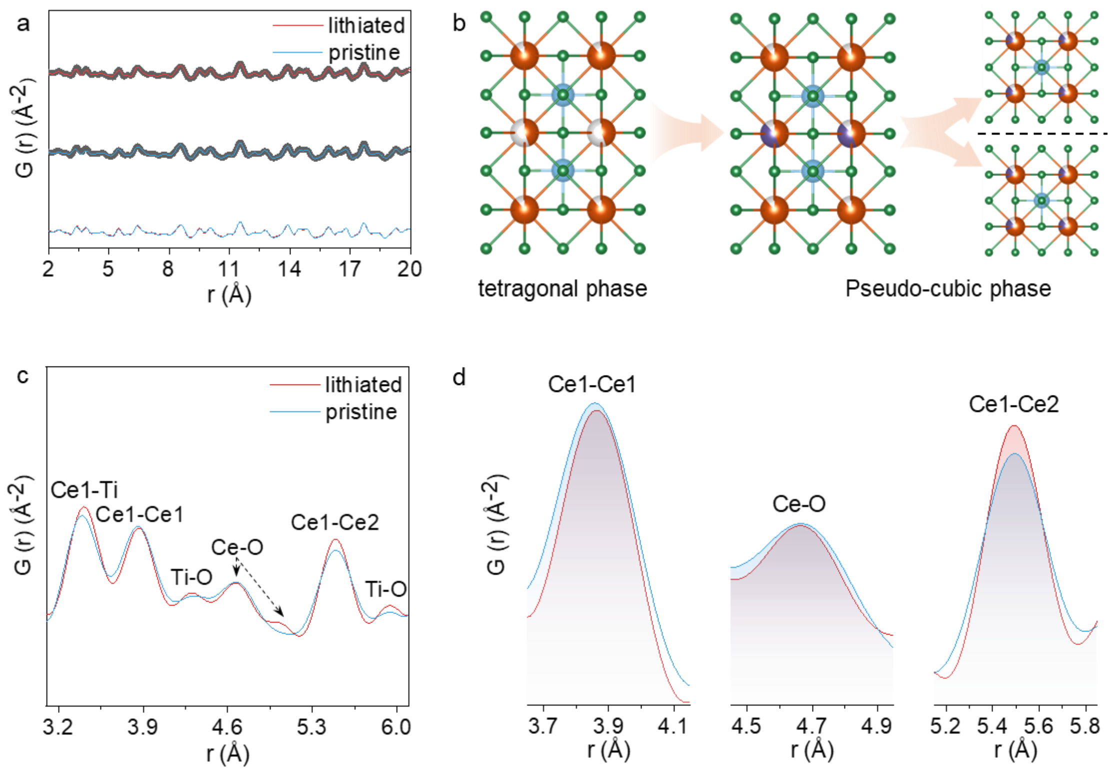


**Supplementary Fig. 30** PDF analysis revealing lithiation-induced transformation from long-range tetragonal ordering to short-range pseudo-cubic ordering. The pristine electrode was examined prior to electrochemical measurements, while the lithiated electrode was harvested after the first discharge to 0.01 V under 0.1 C (25 mA $g^{-1}$) and 25 °C. **a** PDF refinements of pristine and lithiated CTO. **b** Structural models of the long-range ordered tetragonal and short-range ordered pseudo-cubic phases (orange: Ce, blue: Ti, green: O, purple: Li). **c** Comparison of PDF peaks in the short-range region (3.1 - 6.1 Å), where three dominant peaks are assigned to Ce1-Ti1 (~3.4 Å), Ce1-Ce1 (~3.8 Å), and Ce1-Ce2 (~5.5 Å) correlations, additional minor peaks are attributed to Ti-O and Ce-O distances. **d** Comparison of Ce1-Ce1, Ce-O, and Ce1-Ce2 peaks.

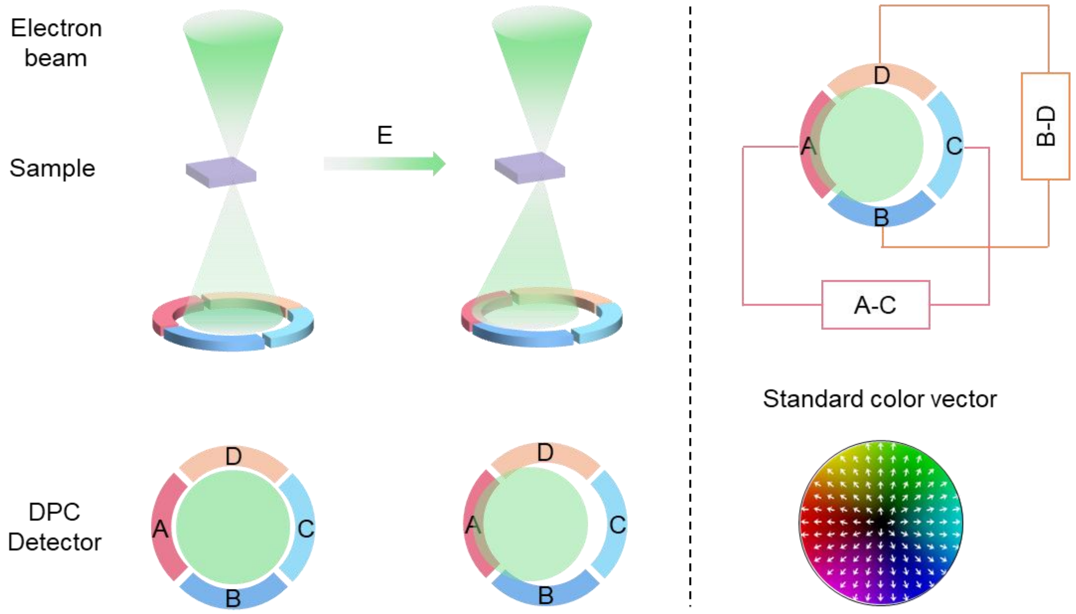


**Supplementary Fig. 31** Schematic illustration the DPC-STEM imaging principle and standard color vector, which can be employed to visualize the direction and distribution of the electric field at the atomic scale.

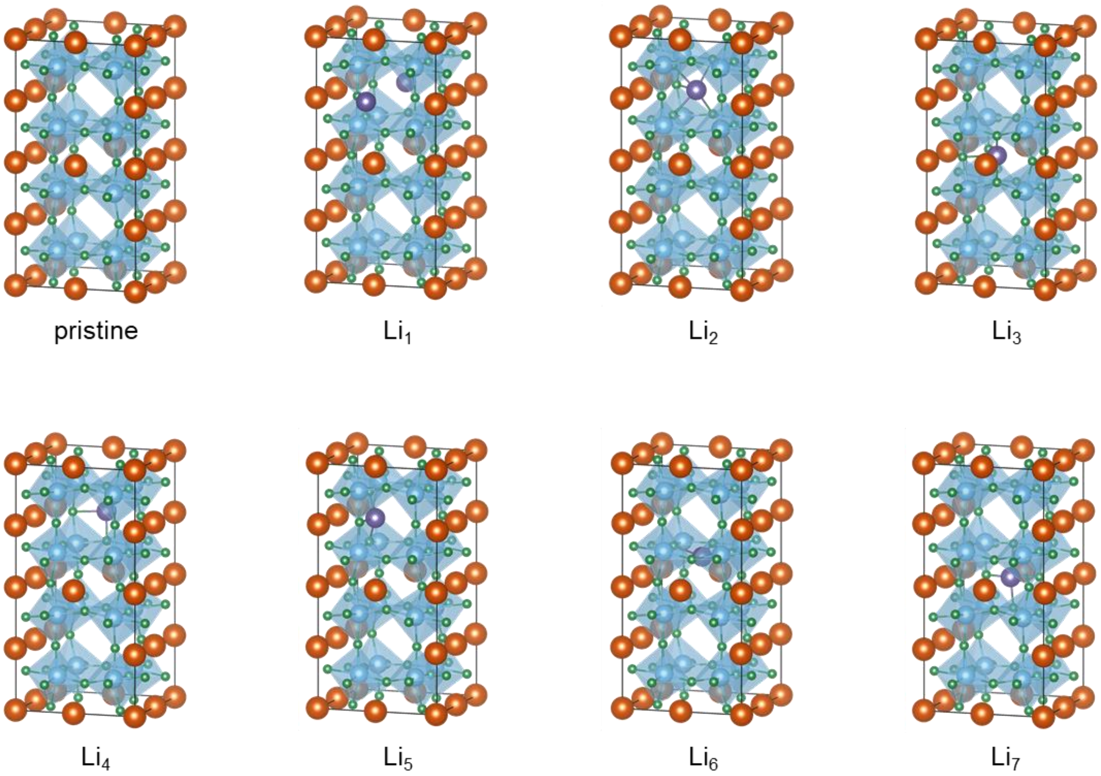


**Supplementary Fig. 32** Optimized structure models depicting potential $Li^{+}$ storage sites in the tetragonal phase (orange: Ce, blue: Ti, green: O, purple: Li).

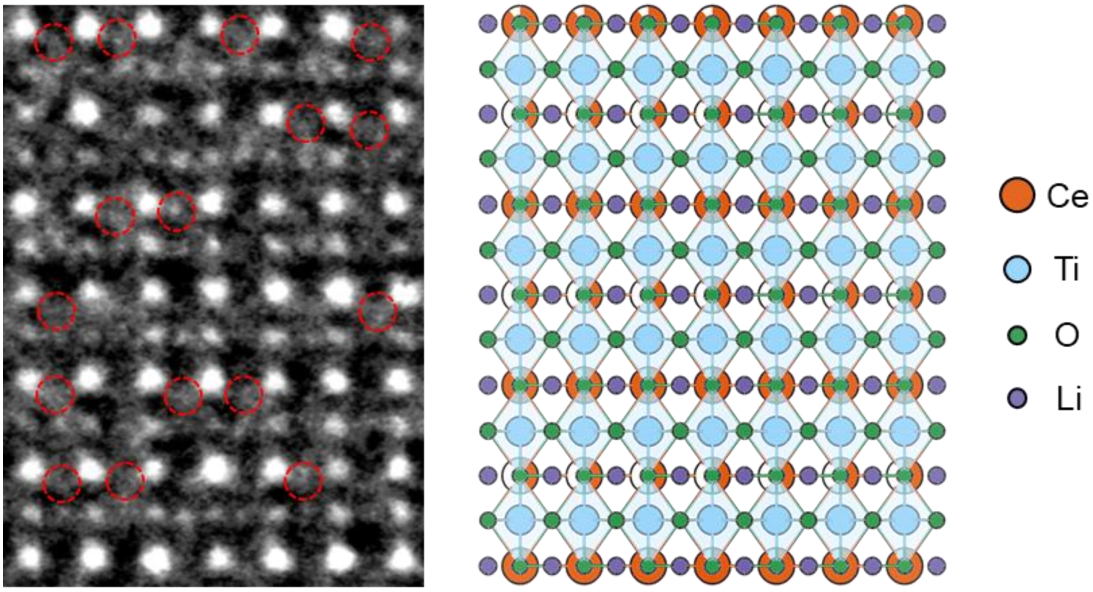


**Supplementary Fig. 33** iDPC-STEM image and corresponding structural model of lithiated CTO along the [110] zone axis, highlighting $Li^+$ insertion into O4 windows between adjacent $TiO_6$ octahedra. The inserted $Li^+$ positions are indicated by red dashed circles. The electrode was collected after the first discharge to 0.01 V under 0.1 C (25 mA $g^{-1}$) and 25 °C.

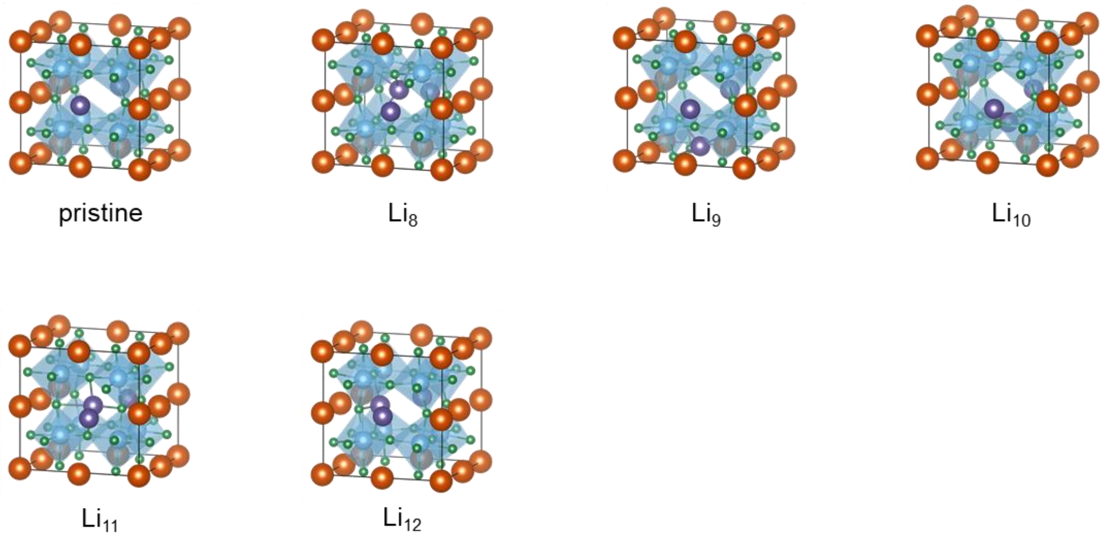


**Supplementary Fig. 34** Optimized structure models depicting potential $Li^{+}$ storage sites in the pseudo-cubic phase (orange: Ce, blue: Ti, green: O, purple: Li).

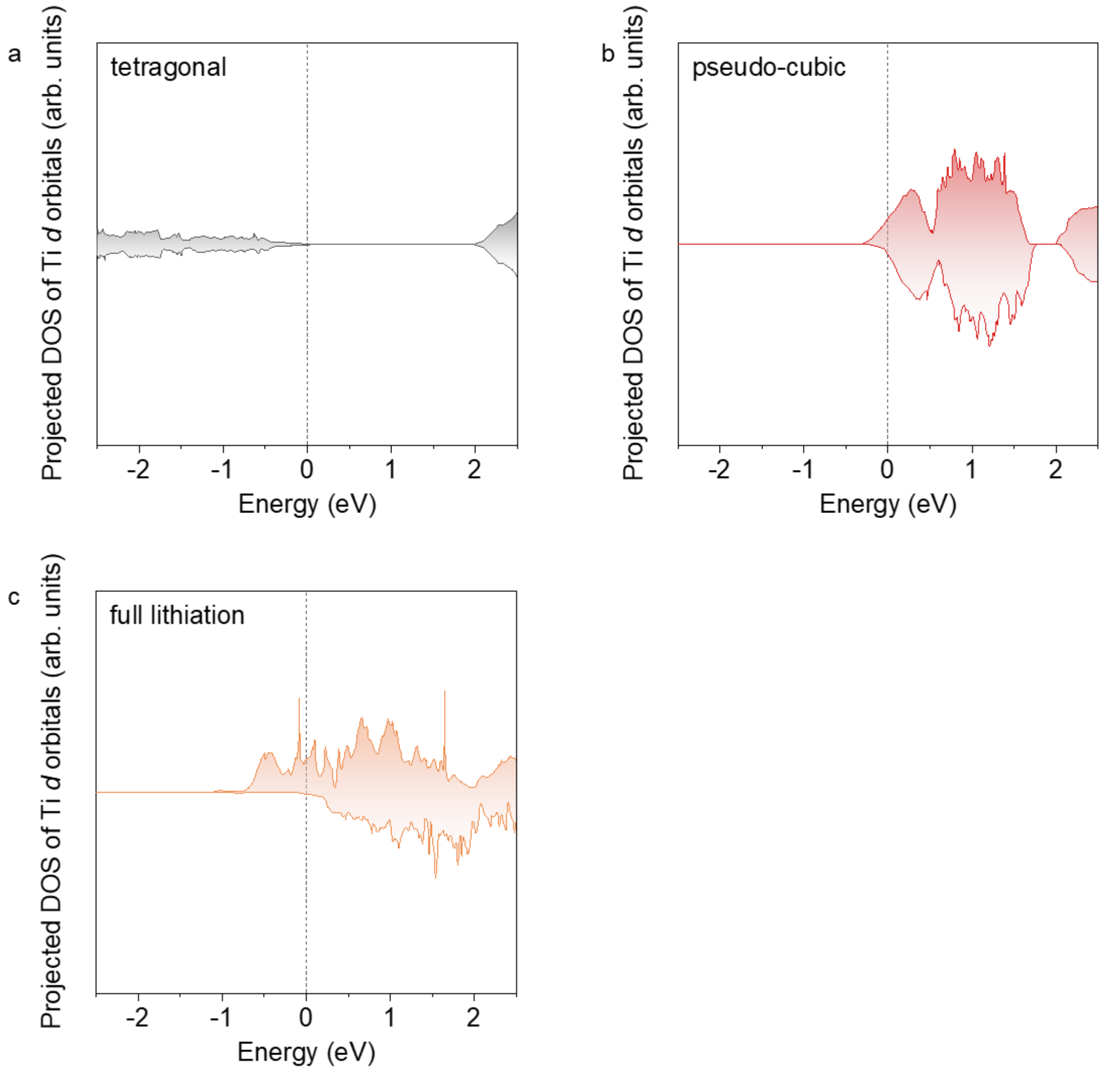


**Supplementary Fig. 35** Projected DOS of Ti *d* orbitals showing the electronic structure evolution upon lithiation. **a** Pristine tetragonal phase. **b** Partially lithiated pseudo-cubic phase. **c.** Fully lithiated CTO.

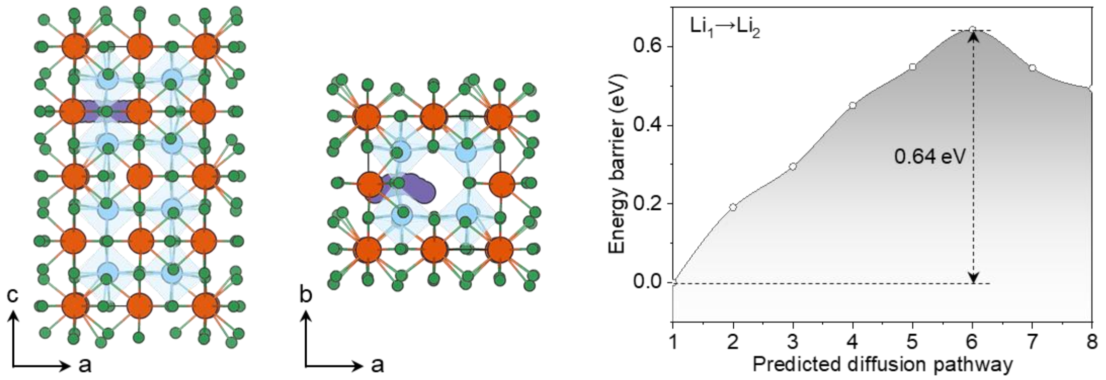


**Supplementary Fig. 36** Predicted $Li^+$ diffusion pathway in the Ce-poor layer of tetragonal CTO and the corresponding energy barrier (orange: Ce, blue: Ti, green: O, purple: Li).

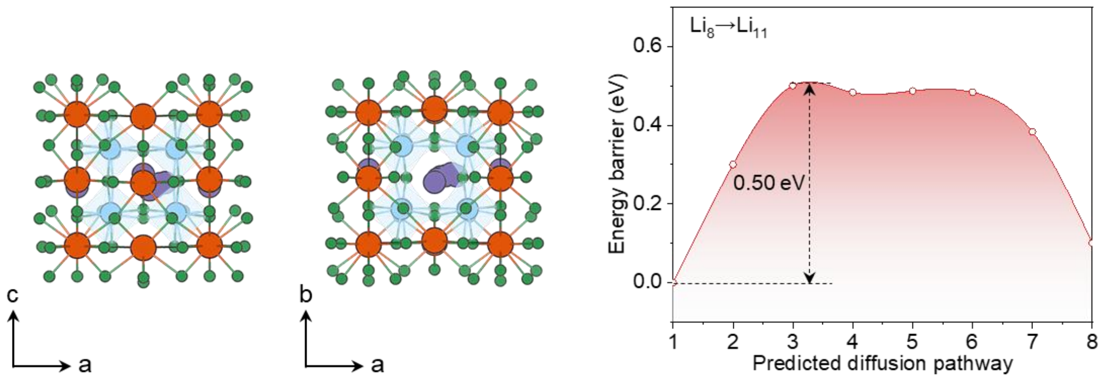


**Supplementary Fig. 37** Predicted $Li^+$ diffusion pathway in the Ce-poor layer of pseudo-cubic CTO and the corresponding energy barrier (orange: Ce, blue: Ti, green: O, purple: Li).

## Supplementary Tables

**Supplementary Table 1** XRD Rietveld refinement results of CTO.

| **Crystal system:** *Tetragonal* **Space group:** *P4/mmm (123)* | | **Lattice parameters:** $a = 3.89537$ Å, $b = 3.89537$ Å, $c = 7.75384$ Å, $\alpha = \beta = \gamma = 90°$ | | **Fitted correlation:** $Rp = 6.7\%$, $Rwp = 9.9\%$, $Chi^2 = 0.94$ | |
|---|---|---|---|---|---|
| **Atom** | **Site** | ***x*** | ***y*** | ***z*** | **Occupancy** |
| Ce1 | *1a* | 0.00000 | 0.00000 | 0.00000 | 0.889 |
| Ce2 | *1b* | 0.00000 | 0.00000 | 0.50000 | 0.444 |
| Ti1 | *2h* | 0.50000 | 0.50000 | 0.26237 | 1.000 |
| O1 | *1c* | 0.50000 | 0.50000 | 0.00000 | 1.000 |
| O2 | *1d* | 0.50000 | 0.50000 | 0.50000 | 1.000 |
| O3 | *4i* | 0.00000 | 0.50000 | 0.26233 | 1.000 |

Refined parameters include lattice parameters ($a$, $b$, $c$), atomic positions ($x$, $y$, $z$), and site occupancies of Ce1 and Ce2. The space group and atomic coordinates of other atoms were fixed during the refinement.

**Supplementary Table 2** Detailed comparison of rate performance among representative negative electrodes.

| Electrodes | Specific current (A g$^{-1}$)<br>Specific capacity (mAh g$^{-1}$) | | | | | | | | Ref. |
|---|---|---|---|---|---|---|---|---|---|
| CTO | 0.025<br>220 | 0.125<br>218 | 0.25<br>206 | 0.5<br>192 | 1.25<br>168 | 2.5<br>147 | 5.0<br>124 | 12.5<br>81 | This work |
| $Li_4Ti_5O_{12}$ | 0.015<br>175 | 0.15<br>125 | 0.45<br>75 | 0.75<br>50 | 1.5<br>35 | | | | 32 |
| Graphite | 0.035<br>375 | 0.07<br>333 | 0.175<br>228 | 0.35<br>73 | 0.7<br>24 | | | | 24 |
| $La_{0.5}Li_{0.5}TiO_3$ | 0.02<br>225 | 0.04<br>175 | 0.1<br>160 | 0.2<br>145 | 0.4<br>125 | 1.0<br>110 | 2.0<br>100 | | 8 |
| T-$Nb_2O_5$ | 0.2<br>150 | 1.0<br>121 | 2.0<br>102 | 6.0<br>67 | | | | | 33 |
| $Li_{0.1}La_{0.3}NbO_3$ | 0.025<br>242 | 0.125<br>186 | 0.25<br>174 | 0.5<br>165 | 1.25<br>153 | 2.5<br>142 | | | 34 |
| $TiNb_2O_7$ | 0.25<br>253 | 0.5<br>226 | 1.25<br>186 | 2.5<br>150 | 5.0<br>105 | 12.5<br>43 | | | 35 |
| $TiO_2$ | 0.0125<br>272 | 0.025<br>254 | 0.05<br>241 | 0.25<br>218 | 1.25<br>148 | 2.5<br>104 | | | 25 |

**Supplementary Table 3** Detailed comparison of cycling performance among representative negative electrodes.

| Electrodes | Current rate (C) | Cycling number | Capacity retention (%) | Ref. |
|---|---|---|---|---|
| CTO | 20 | 10000 | 82.5 | This work |
| $La_{0.5}Li_{0.5}TiO_3$ | 10 | 3000 | 79 | [8] |
| $NiNb_2O_6$ | 10 | 1000 | 92.8 | [9] |
| $Li_3VO_4$@C | 10 | 1000 | 95 | [10] |
| $SrVO_3$ | 2 | 2500 | 80 | [11] |
| T-$Nb_2O_5$@C | 2 | 2000 | 87.9 | [12] |
| $Li_4Ti_5O_{12}$ | 10 | 1000 | 90 | [13] |
| $Nb_{16}W_5O_{55}$ | 20 | 750 | 95 | [14] |
| $TiNb_2O_7$ | 10 | 900 | 80.6 | [15] |
| $CeNb_3O_9$ | 10 | 2000 | 89.1 | [16] |
| $LiYTiO_4$ | 20 | 3000 | 98 | [17] |
| $Li_{0.35}Nd_{0.55}TiO_3$ | 10 | 1600 | 93.5 | [18] |
| $Li_{0.38}Pr_{0.54}TiO_3$ | 10 | 2100 | 96.75 | [19] |

**Supplementary Table 4** Detailed comparison of $Li^+$ diffusion coefficients among representative negative electrodes.

| Electrodes | Lithiation diffusion coefficient ($cm^2$ $s^{-1}$) | Delithiation diffusion coefficient ($cm^2$ $s^{-1}$) | Ref. |
|---|---|---|---|
| CTO | $5.29 \times 10^{-11}$ | $9.39 \times 10^{-11}$ | This work |
| $CeNb_3O_9$ | $\sim 10^{-12}$ | $\sim 10^{-12}$ | 16 |
| $TiNb_2O_7$ | $1.4 \times 10^{-13}$ | $1.6 \times 10^{-13}$ | 29 |
| $Li_4Ti_5O_{12}$ | $1.469 \times 10^{-12}$ | $3.295 \times 10^{-12}$ | 36 |
| $Nb_{18}W_{16}O_{93}$ | $1.1 \times 10^{-13}$ | $1.1 \times 10^{-13}$ | 14 |
| $Li_{0.38}Pr_{0.54}TiO_3$ | $1.08 \times 10^{-12}$ | $1.27 \times 10^{-12}$ | 19 |
| $Li_{0.35}Nd_{0.55}TiO_3$ | $2.07 \times 10^{-11}$ | $2.78 \times 10^{-11}$ | 18 |
| $Li_3VO_4$ | $1.084 \times 10^{-12}$ | $1.084 \times 10^{-12}$ | 37 |
| $Li_3V_2O_5$ | $5.02 \times 10^{-14}$ | $5.02 \times 10^{-14}$ | 38 |
| $Nb_2O_5$ | $3.74 \times 10^{-13}$ | $7.64 \times 10^{-14}$ | 39 |
| $NiNb_2O_6$ | $1.20 \times 10^{-12}$ | $1.36 \times 10^{-12}$ | 40 |
| $Ti_2Nb_{10}O_{29}$ | $3.0 \times 10^{-14}$ | $4.8 \times 10^{-13}$ | 41 |

**Supplementary Table 5** Formation energies of different $Li^+$ storage sites in the tetragonal phase.

| **$Li^+$ storage sites** | **Free energy (eV)** | **Formation energy (eV)** |
|---|---|---|
| Pristine | -605.49906117 | 0.00000000 |
| $Li_1$ | -611.85515242 | -4.45219713 |
| $Li_2$ | -611.36189135 | -3.95893606 |
| $Li_3$ | -611.77873128 | -4.37577599 |
| $Li_4$ | -611.82169986 | -4.41874457 |
| $Li_5$ | -611.44550103 | -4.04254574 |
| $Li_6$ | -611.75829348 | -4.35533819 |
| $Li_7$ | -611.71583378 | -4.31287849 |

**Supplementary Table 6** Formation energies of different $Li^+$ storage sites in the pseudo-cubic phase.

| $Li^+$ storage sites | Free energy (eV) | Formation energy (eV) |
|---|---|---|
| Pristine | -308.48216030 | 0.00000000 |
| $Li_8$ | -311.98629707 | -1.60024265 |
| $Li_9$ | -312.08157747 | -1.69552305 |
| $Li_{10}$ | -311.92746288 | -1.54140846 |
| $Li_{11}$ | -311.88556822 | -1.49951380 |
| $Li_{12}$ | -311.78662127 | -1.40056685 |

## Supplementary References


1. Tran Huu H., Vu N. H., Ha H., Moon J., Kim H. Y., Im W. B. Sub-micro droplet reactors for green synthesis of $Li_3VO_4$ anode materials in lithium ion batteries. *Nat. Commun.* **12**, 3081 (2021).
2. Wu D., *et al.* In Situ Fabrication of Graphdiyne Nanoisland Anchored $Ti_3C_2T_x$ Film to Accelerate Intercalation Pseudocapacitance Kinetics. *Adv. Energy Mater.* **14**, 2304404 (2024).
3. van den Bergh W., Lokupitiya H. N., Vest N. A., Reid B., Guldin S., Stefik M. Nanostructure Dependence of T-$Nb_2O_5$ Intercalation Pseudocapacitance Probed Using Tunable Isomorphic Architectures. *Adv. Funct. Mater.* **31**, 2007826 (2021).
4. Pu X., *et al.* Understanding and Calibration of Charge Storage Mechanism in Cyclic Voltammetry Curves. *Angew. Chem. Int. Ed.* **60**, 21310-21318 (2021).
5. Peng B., *et al.* Toward Extremely Fast Charging through Boosting Intercalative Redox Pseudocapacitance: A $SbCrSe_3$ Anode for Large and Fast Sodium Storage. *Adv. Energy Mater.* **13**, 2203187 (2023).
6. Zhang S., *et al.* Dehydration-Triggered Ionic Channel Engineering in Potassium Niobate for Li/K-Ion Storage. *Adv. Mater.* **32**, 2000380 (2020).
7. Jing P., *et al.* Tailoring the Wadsley–Roth crystallographic shear structures for high-power lithium-ion batteries. *Energy Environ. Sci.* **17**, 6571-6581 (2024).
8. Zhang L., *et al.* Lithium lanthanum titanate perovskite as an anode for lithium ion batteries. *Nat. Commun.* **11**, 3490 (2020).
9. Zhao Y., *et al.* “Zero-Strain” $NiNb_2O_6$ Fibers for All-Climate Lithium Storage. *Nano-Micro Lett.* **17**, 15 (2024).
10. Shen L., Chen S., Maier J., Yu Y. Carbon-Coated $Li_3VO_4$ Spheres as Constituents of an Advanced Anode Material for High-Rate Long-Life Lithium-Ion Batteries. *Adv. Mater.* **29**, 1701571 (2017).
11. Li X., *et al.* Perovskite-Type $SrVO_3$ as High-Performance Anode Materials for Lithium-Ion Batteries. *Adv. Mater.* **34**, 2107262 (2022).
12. Meng J., *et al.* Identification of Phase Control of Carbon-Confined $Nb_2O_5$ Nanoparticles toward High-Performance Lithium Storage. *Adv. Energy Mater.* **9**, 1802695 (2019).
13. Kim H.-m., *et al.* Mixed anion effects on structural and electrochemical characteristics of $Li_4Ti_5O_{12}$ for high-rate and durable anode materials. *J. Mater. Chem. A* **12**, 7107-7121 (2024).
14. Griffith K. J., Wiaderek K. M., Cibin G., Marbella L. E., Grey C. P. Niobium tungsten oxides for high-rate lithium-ion energy storage. *Nature* **559**, 556-563 (2018).
15. Yu H., *et al.* $TiNb_2O_7$ hollow nanofiber anode with superior electrochemical performance in rechargeable lithium ion batteries. *Nano Energy* **38**, 109-117 (2017).

16. Yang L., *et al.* Atomic Short-Range Order in a Cation-Deficient Perovskite Anode for Fast-Charging and Long-Life Lithium-Ion Batteries. *Adv. Mater.* **34**, 2200914 (2022).
17. Zhang Y., *et al.* Layered Perovskite Lithium Yttrium Titanate as a Low-Potential and Ultrahigh-Rate Anode for Lithium-Ion Batteries. *Adv. Energy Mater.* **12**, 2200922 (2022).
18. Liu H., *et al.* Cation-deficient perovskite $Li_{0.35}Nd_{0.55}TiO_3$ as a high-performance anode for lithium-ion batteries. *Nano Energy* **119**, 109065 (2024).
19. Liu H., *et al.* A-site deficient perovskite lithium praseodymium titanate as a high-rate anode for lithium-ion batteries. *Chem. Eng. J.* **479**, 147765 (2024).
20. Prakash A. S., Manikandan P., Ramesha K., Sathiya M., Tarascon J. M., Shukla A. K. Solution-Combustion Synthesized Nanocrystalline $Li_4Ti_5O_{12}$ As High-Rate Performance Li-Ion Battery Anode. *Chem. Mater.* **22**, 2857-2863 (2010).
21. Wang C., *et al.* Combining Fast Li-Ion Battery Cycling with Large Volumetric Energy Density: Grain Boundary Induced High Electronic and Ionic Conductivity in $Li_4Ti_5O_{12}$ Spheres of Densely Packed Nanocrystallites. *Chem. Mater.* **27**, 5647-5656 (2015).
22. Zhang N., Liu Z., Yang T., Liao C., Wang Z., Sun K. Facile preparation of nanocrystalline $Li_4Ti_5O_{12}$ and its high electrochemical performance as anode material for lithium-ion batteries. *Electrochem. Commun.* **13**, 654-656 (2011).
23. Jung H.-G., *et al.* Microscale spherical carbon-coated $Li_4Ti_5O_{12}$ as ultra high power anode material for lithium batteries. *Energy Environ. Sci.* **4**, 1345-1351 (2011).
24. Billaud J., Bouville F., Magrini T., Villevieille C., Studart A. R. Magnetically aligned graphite electrodes for high-rate performance Li-ion batteries. *Nat. Energy* **1**, 16097 (2016).
25. Hu Y. S., Kienle L., Guo Y. G., Maier J. High Lithium Electroactivity of Nanometer-Sized Rutile $TiO_2$. *Adv. Mater.* **18**, 1421-1426 (2006).
26. Jiang C., Wei M., Qi Z., Kudo T., Honma I., Zhou H. Particle size dependence of the lithium storage capability and high rate performance of nanocrystalline anatase $TiO_2$ electrode. *J. Power Sources* **166**, 239-243 (2007).
27. Liu H., *et al.* Mesoporous $TiO_2$–B Microspheres with Superior Rate Performance for Lithium Ion Batteries. *Adv. Mater.* **23**, 3450-3454 (2011).
28. Liu M., Yan C., Zhang Y. Fabrication of $Nb_2O_5$ Nanosheets for High-rate Lithium Ion Storage Applications. *Sci. Rep.* **5**, 8326 (2015).
29. Zhang Y., *et al.* Delocalized electronic engineering of $TiNb_2O_7$ enables low temperature capability for high-areal-capacity lithium-ion batteries. *Nat. Commun.* **15**, 6299 (2024).
30. Augustyn V., *et al.* High-rate electrochemical energy storage through Li+ intercalation pseudocapacitance. *Nat. Mater.* **12**, 518-522 (2013).
31. Patterson A. R., *et al.* Rapid and Reversible Lithium Insertion in the Wadsley–Roth-Derived Phase $NaNb_{13}O_{33}$. *Chem. Mater.* **35**, 6364-6373 (2023).

32. Haridas A. K., Sharma C. S., Rao T. N. Donut-Shaped $Li_4Ti_5O_{12}$ Structures as a High Performance Anode Material for Lithium Ion Batteries. *Small* **11**, 290-294 (2015).
33. Griffith K. J., Forse A. C., Griffin J. M., Grey C. P. High-Rate Intercalation without Nanostructuring in Metastable $Nb_2O_5$ Bronze Phases. *J. Am. Chem. Soc.* **138**, 8888-8899 (2016).
34. Xiong X*., et al.* A Low Strain A-Site Deficient Perovskite Lithium Lanthanum Niobate Anode for Superior $Li^+$ Storage. *Adv. Funct. Mater.* **32**, 2106911 (2022).
35. Wu W*., et al.* Unprecedented Superhigh-Rate and Ultrastable Anode for High-Power Battery via Cationic Disordering. *Adv. Energy Mater.* **12**, 2201130 (2022).
36. Yan B*., et al.* Novel understanding of carbothermal reduction enhancing electronic and ionic conductivity of $Li_4Ti_5O_{12}$ anode. *J. Mater. Chem. A* **3**, 11773-11781 (2015).
37. Yang G*., et al.* Morphology controlled lithium storage in $Li_3VO_4$ anodes. *J. Mater. Chem. A* **6**, 456-463 (2018).
38. Ren Z., Yu S., Yao T., Xu T., He J., Shen L. Crystal phase and morphology engineering of ω-$Li_3V_2O_5$ nanospheres for high-rate lithium-ion capacitors. *J. Mater. Chem. A* **11**, 621-629 (2023).
39. Zheng Y., Yao Z., Shadike Z., Lei M., Liu J., Li C. Defect-Concentration-Mediated T-$Nb_2O_5$ Anodes for Durable and Fast-Charging Li-Ion Batteries. *Adv. Funct. Mater.* **32**, 2107060 (2022).
40. Xia R*., et al.* Nickel Niobate Anodes for High Rate Lithium-Ion Batteries. *Adv. Energy Mater.* **12**, 2102972 (2022).
41. Yuan T*., et al.* A hierarchical $Ti_2Nb_{10}O_{29}$ composite electrode for high-power lithium-ion batteries and capacitors. *Mater. Today* **45**, 8-19 (2021).
