# Supplementary material for: Vacancy-ordered perovskite superlattice in cerium titanate negative electrode for enhanced lithium-ion storage
Source: Nat Commun. 2025 Dec 12;16:11413. doi: 10.1038/s41467-025-66233-6 (PMC12738579; doi:10.1038/s41467-025-66233-6)
Supplement: Supplementary file 3 — Description of Additional Supplementary Files [file 41467_2025_66233_MOESM3_ESM.docx]

**Description of Additional Supplementary Files**

**Supplementary Data 1.** Optimized structure model of Ce_10_Ti_16_O_48_.

**Supplementary Data 2.** Optimized structure model of Li_2_Ce_5_Ti_8_O_24_.

**Supplementary Data 3.** Optimized structure model of Li_5_Ce_5_Ti_8_O_24_.
